# Material-Efficient Selection of Externally Bonded Fiber-Reinforced Polymer Composites for RC Shear Strengthening Using a Literature-Based Database and Break-Even Criteria

**DOI:** 10.3390/polym18202503

**Published:** 2026-10-13

**Authors:** Nageh M. Ali, Ahmed Farghaly

**Affiliations:** Department of Civil and Environmental Engineering, College of Engineering, Majmaah University, Al-Majmaah 11952, Saudi Arabia; am.farghaly@mu.edu.sa

**Keywords:** FRP shear strengthening, material efficiency, greenhouse-gas break-even, polymer composites, reinforced concrete, externally bonded FRP, sustainable strengthening

## Abstract

Fiber-reinforced polymer (FRP) shear strengthening is generally selected on the basis of structural capacity, whereas the efficiency with which installed material contributes to shear resistance has received little quantitative attention. This study develops a literature-based material-efficiency framework from a database of 359 published tests compiled from 43 experimental programs covering carbon, glass, aramid, basalt, and PET-FRP systems. Material efficiency (ME) was defined as the experimentally attributed FRP shear contribution per unit mass of installed composite sheet. ME varied approximately 30-fold. Median ME was 303 kN·m·kg^−1^ for CFRP and 38 kN·m·kg^−1^ for GFRP, giving a CFRP:GFRP median-efficiency ratio of 8.1 (program-clustered 95% CI: 3.8–18.3; 2.8–21.3 when thickness-convention and density uncertainty were also propagated). This observational, database-level ratio, which is an association rather than a causal material effect, was translated into a sheet-level cradle-to-gate greenhouse-gas break-even screening criterion. A response-surface model based on XGBoost achieved random ten-fold out-of-fold R^2^ = 0.802, decreasing to 0.526 under program-grouped validation and 0.394 under leave-one-program-out validation; most of this loss reflected program-level offsets. Member geometry and transverse reinforcement carried greater predictive importance for the attributed FRP contribution than FRP-sheet properties under both random and program-grouped validation. Material efficiency generally decreased with increasing installed sheet rigidity, consistent with diminishing structural returns from additional FRP material. The framework compares code-compliant alternatives that satisfy the required FRP shear contribution, using material efficiency to identify lower-material-demand solutions before project-specific environmental and economic evaluation.

## 1. Introduction

Strengthening structurally deficient reinforced-concrete members rather than replacing them can substantially reduce material consumption, preserve the embodied impacts of the existing structure, and avoid demolition-related waste [1,2]. Among available strengthening technologies, externally bonded fiber-reinforced polymer composites have become widely used for shear upgrading because of their high specific strength, corrosion resistance, low self-weight, and ease of installation [3,4]. Designers may select among carbon, glass, aramid, basalt, and large-rupture-strain PET fibers; side-bonded, U-jacketed, and completely wrapped configurations; and anchored or unanchored systems. These alternatives differ markedly in structural efficiency, material demand, and cost, with corresponding environmental implications. Nevertheless, selection is still governed predominantly by strength and detailing requirements, with limited quantitative guidance concerning the shear contribution obtained per unit mass of installed composite.

Two adjacent bodies of literature approach this problem without resolving it fully. The first concerns data-driven material optimization, including approaches coupling mechanical performance with cost or greenhouse-gas objectives [5,6,7,8]. These studies primarily optimize materials for new construction rather than strengthening systems for existing members [9,10,11]. The second concerns life-cycle assessment of composite construction and retrofit systems. Previous studies have quantified cradle-to-gate burdens of CFRP and evaluated environmental performance of composite structural and retrofit systems [2,12,13]. These assessments remain tied to specific structures, functional units, product inventories, and assumed emission factors and do not establish a database-wide relationship between delivered FRP shear contribution and installed composite mass across different FRP systems and strengthening configurations.

The mechanics of externally bonded FRP shear strengthening further complicate material selection. The mobilized FRP contribution is controlled by competing rupture and debonding mechanisms, and current design approaches differ in their treatment of effective strain and bond limitations [14]. Debonding commonly governs side-bonded and U-jacketed systems because interfacial fracture localizes over a finite effective bond length; consequently, additional bonded length or sheet thickness beyond the effective range may add material without producing a proportional capacity increase [15]. The effective FRP strain is therefore determined not only by fiber strength and stiffness but also by member geometry, crack kinematics, concrete conditions, internal reinforcement, and anchorage.

Analytical and experimental evidence indicates that internal transverse reinforcement and externally bonded FRP do not contribute independently to shear resistance. Spinella [16] showed that external FRP strengthening can reduce the stirrup stress mobilized at failure. Experimental strain measurements have shown that externally applied FRP and FRCM strengthening systems interact with crack development and internal transverse-reinforcement engagement [17]. Anchorage systems can delay debonding and increase the mobilized strain of an already installed sheet, often with only a limited increase in composite mass [4,18,19]. These findings collectively indicate that the delivered FRP contribution emerges from the response of the strengthened member–bond system rather than from the composite sheet alone.

Although state-of-the-art reviews document the structural performance of FRP strengthening systems extensively, they provide limited resource-based comparison of the shear resistance delivered per unit of installed composite [3,20]. Conventional performance indicators emphasize ultimate capacity, strengthening ratio, or predicted FRP contribution. Such indicators do not distinguish between two systems that deliver similar capacity increases while requiring substantially different quantities of composite material. A resource-normalized metric is therefore needed to identify designs that achieve the required structural contribution with lower installed material demand.

Data-driven methods have been applied increasingly to FRP- and fabric-reinforced cementitious matrix (FRCM)-strengthened concrete members, including prediction of shear resistance, flexural capacity, and failure mode [21]. Ensemble tree methods, including random forests, CatBoost, and XGBoost, have shown high reported predictive performance on structural-concrete datasets [9,22,23]. However, two methodological issues warrant particular attention.

First, predictive performance is frequently evaluated using random train–test splitting. When multiple specimens originate from the same experimental program, random splitting can place closely related beams in both the training and test sets. The resulting accuracy may therefore reflect within-program interpolation rather than transfer to an independent experimental program [9,23,24].

Second, interpretation of predictive models often relies on a single attribution method, most commonly SHAP. Feature-credit allocation can be unstable when predictors are correlated or redundant [25]. Accordingly, complementary attribution methods are required to distinguish marginal association, predictive dependence, unique information, and interaction effects [26,27,28]. Once structural efficiency has been quantified, environmental comparison of FRP systems is also sensitive to methodological assumptions. Published FRP emission factors vary substantially with fiber type, product form, resin content, electricity mix, manufacturing route, and system boundary [29,30,31]. This heterogeneity makes fixed-factor environmental rankings context-dependent. A more transferable approach is therefore to separate the database-derived structural-performance ratio from the project-specific environmental-factor ratio. The structural database can provide the material-efficiency break-even threshold, while the designer supplies the relevant emission-factor ratio, project-appropriate, boundary-compatible environmental inventories or product declarations.

The present work differs from previous studies in four respects. First, existing FRP shear databases and machine-learning studies predict shear capacity or the FRP contribution [21,22,23,24], and experimental reviews compare structural performance [1,3,20]; systematic normalization of the delivered contribution by the installed composite mass has received comparatively limited attention. The present study operationalizes material efficiency as this normalization and quantifies it across fiber types, wrapping schemes, section types, and anchorage conditions. Second, instead of ranking materials with fixed emission factors or prices, which are context-dependent [12,13,29,30,31], the study derives a break-even threshold from the structural data and leaves the emission-factor or price ratio to the designer. Third, program-grouped validation, leave-one-program-out validation, and program-clustered bootstrap intervals are used throughout, so that the strength of the evidence is not overstated by within-program interpolation. Fourth, the study is a quantitative synthesis of published experimental results rather than a new experimental program: no new tests were performed, every source program is cited in the reference list and characterized in Appendix A, and its contribution therefore lies in the metric, the decision framework, and the transparent treatment of cross-program heterogeneity that a single test series cannot provide.

The present study develops a literature-based framework for the material-efficient selection of externally bonded FRP shear-strengthening systems. A curated database of 359 published tests was used to achieve the following: (1) define and quantify material efficiency as the experimentally attributed FRP shear contribution per unit mass of installed composite sheet; (2) compare the resulting efficiency distributions across fiber types, wrapping schemes, section types, and anchorage conditions; (3) derive greenhouse-gas and cost break-even criteria that depend on database-derived performance ratios rather than assumed universal emission factors or prices; (4) develop a response-surface surrogate based on XGBoost for the attributed FRP shear contribution; (5) evaluate predictive performance under random, program-grouped, and leave-one-program-out validation; and (6) interpret the response using complementary attribution methods and density-bounded efficiency maps. The proposed framework complements, rather than replaces, code-based FRP shear design by supporting material-efficient selection among structurally admissible strengthening alternatives while enabling subsequent environmental and economic comparisons.

## 2. Database and Efficiency Metrics

### 2.1. Database

The present database spans five fiber types, three externally bonded wrapping schemes, rectangular and flanged sections, anchored and unanchored systems, and both intact and pre-cracked members. The records were compiled from 45 published sources reported between 1997 and 2026 [32,33,34,35,36,37,38,39,40,41,42,43,44,45,46,47,48,49,50,51,52,53,54,55,56,57,58,59,60,61,62,63,64,65,66,67,68,69,70,71,72,73,74,75,76], 44 of which are experimental programs that contributed specimen records. No new tests were performed in this study. Every source program is cited in the reference list and characterized in Table A1 (Appendix A), which gives for each program the numbers of retained and excluded specimens and the fiber types, wrapping schemes, section types, anchorage, and pre-cracking conditions represented; the specimen-level records, with their source attribution, are provided in the Supplementary Materials. Of the 376 collected specimens, 359 exhibited a positive FRP-attributed increase in shear capacity, denoted *V_f_*_,exp_, and were retained for the primary analysis. These specimens originated from 43 experimental programs and included 291 carbon-FRP, 37 glass-FRP, 14 aramid-FRP, seven basalt-FRP, and 10 large-rupture-strain PET-FRP systems. The database further comprised 139 side-bonded, 159 U-jacketed, and 61 completely wrapped configurations; 210 rectangular and 149 T-section beams; 90 anchored systems; and 46 pre-cracked members. This breadth enables comparative material-efficiency analysis while introducing cross-program heterogeneity [21,77]. To make the scope of the database concrete, Table A2 presents a representative strengthening scheme for each principal combination of fiber type, wrapping scheme, section type, anchorage, and pre-cracking, selected as the specimen whose material efficiency was closest to the median of its category.

For each strengthened specimen, the attributed FRP contribution was taken as the difference between the strengthened and companion unstrengthened capacities,

(1) *V_f_*_,exp_ = *V*_strengthened_ − *V*_control_

This definition isolates the incremental shear contribution attributed to the strengthening system rather than the total shear resistance. The remaining 17 specimens were excluded because their attributed FRP contribution was zero, negative, or undetermined; all are listed in Table S11 of the Supplementary Materials. Because the principal material-efficiency analysis is conducted on a logarithmic scale, non-positive values of *V_f_*_,exp_ cannot be represented. Their influence was evaluated in two sensitivity analyses reported in Section 4.2: all excluded specimens were reintroduced as zero-contribution observations, and the nine measurable non-positive values were retained with their signed efficiencies.

### 2.2. Material-Efficiency Metric and Scope

The functional unit was one meter of strengthened beam length. For each specimen, the installed FRP sheet mass per meter of strengthened zone was calculated from the reported sheet thickness, number of plies, strip continuity, bonded perimeter, and nominal composite density.

The bonded perimeter, *p*, was defined according to the wrapping configuration as

(2) $$p=\{\begin{array}{cc} 2d_{\mathrm{fv}}, & \mathrm{side}-\mathrm{bonded}\\ 2d_{\mathrm{fv}}+b_{w}, & U-\mathrm{jacketed}\\ 2(h+b_{w}), & \mathrm{completely wrapped} \end{array}$$

where *d_fv_* is the effective FRP depth, *b_w_* is the web width, and *h* is the total section depth.

The nominal composite-sheet mass per meter, *m*, was then calculated as

(3) $$m=t_{f}\;n\;\left(\frac{w_{f}}{s_{f}}\right)p\;\rho_{\mathrm{FRP}}\;10^{-3}$$

where *t_f_* is the nominal thickness of one FRP ply, *n* is the number of plies, *w_f_*/*s_f_* is the strip-continuity ratio, and *ρ_FRP_* is the nominal density of the cured composite. Nominal densities of 1.60, 2.00, 1.40, 2.10, and 1.35 g cm^−3^ were assigned to carbon fiber-reinforced polymer (CFRP), glass fiber-reinforced polymer (GFRP), aramid fiber-reinforced polymer (AFRP), basalt fiber-reinforced polymer (BFRP), and polyethylene terephthalate fiber-reinforced polymer (PET-FRP) systems, respectively. These values represent typical cured composite products rather than dry fibers. The source programs do not report sheet thickness using a uniform convention; reported thicknesses may correspond to dry-fiber-equivalent thickness, fabric thickness, cured-laminate thickness, or manufacturer-design thickness. Combining these thickness conventions with a common cured-composite density introduces additional uncertainty into the material-efficiency metric. The reported efficiencies should therefore be interpreted as harmonized nominal estimates for cross-study comparison rather than specimen-specific measurements of installed composite mass. This convention uncertainty was propagated explicitly into the principal break-even ratio (Section 3.3). Fiber areal weight, weave architecture, manufacturing route (wet lay-up or pre-cured), and fiber volume fraction were also inconsistently reported and could not be controlled individually. Their associated variability is partly reflected in the reported sheet properties of each specimen (thickness, *E_f_*, and *f_fu_*), through the within-material scatter of ME reported in Table 1, and through the program-clustered bootstrap, which treats the materials and fabrication of each program as a shared cluster effect.

Material efficiency was defined as

(4) $$\mathrm{ME}=\frac{V_{f,\exp}}{m}$$

with units of kN·m·kg^−1^. The metric represents the experimentally attributed FRP shear contribution delivered per unit mass of installed composite sheet. Three system-boundary decisions were considered:

First, the mass term includes the cured FRP composite sheet only. Adhesive, primer, putty, and other installation consumables are excluded because their quantities were not reported consistently. Second, the environmental boundary is the cradle-to-gate production of the cured composite sheet. Transport, installation energy, labor, maintenance, replacement, and end-of-life treatment are excluded. Third, the metric inherits the uncertainty of the experimentally attributed contribution. Accordingly, the analysis emphasizes medians, interquartile ranges, bootstrap confidence intervals, and density-bounded decision maps rather than deterministic point estimates.

### 2.3. Break-Even Formulation

Environmental comparison was formulated as a break-even problem rather than through fixed emission factors. This choice reflects the substantial variability in published FRP inventories, arising from differences in product definition, manufacturing, and system boundary [12,30,31].

Let *EF_i_* denote the cradle-to-gate greenhouse-gas emission factor of system *i*, expressed in kg CO_2_ e per kilogram of cured composite sheet. The greenhouse-gas efficiency of system *i* may be written as

(5) $$GE_{i}=\frac{ME_{i}}{EF_{i}}$$

where *ME_i_* is the material efficiency of that system.

Consider two systems, *A* and *B*. System *B* is the lower-GHG option per unit of delivered FRP shear contribution when

(6) $$\frac{ME_{B}}{EF_{B}}>\frac{ME_{A}}{EF_{A}}$$

Rearranging gives

(7) $$\frac{EF_{A}}{EF_{B}}>\frac{ME_{A}}{ME_{B}}$$

The right-hand side is the material-efficiency ratio derived from the published experimental database, whereas the left-hand side is the project-specific emission-factor ratio obtained from boundary-compatible environmental inventories or product declarations. The material-efficiency ratio therefore defines the break-even threshold.

For the CFRP-to-GFRP comparison, the decision rule becomes

(8) $$\frac{EF_{\mathrm{CFRP}}}{EF_{\mathrm{GFRP}}}>\frac{ME_{\mathrm{CFRP}}}{ME_{\mathrm{GFRP}}}$$

If the emission-factor ratio exceeds the measured material-efficiency ratio, GFRP has the lower sheet-level cradle-to-gate GHG burden per unit of delivered shear contribution under the adopted boundary. Otherwise, CFRP has the lower corresponding burden.

The same formulation applies to cost. Let *P_i_* denote the price per kilogram of system *i*. The cost efficiency is

(9) $$CE_{i}=\frac{ME_{i}}{P_{i}}$$

and system *B* is more cost-efficient than system *A* when

(10) $$\frac{P_{A}}{P_{B}}>\frac{ME_{A}}{ME_{B}}$$

The database-derived material-efficiency ratio therefore provides a common structural threshold for environmental and economic comparisons while remaining separate from project-specific emission factors and prices.

## 3. Methods

### 3.1. Response-Surface Model

An XGBoost response-surface model was developed to predict the experimentally attributed FRP shear contribution, *V_f_*_,exp_. XGBoost was selected because boosted decision trees perform effectively on small-to-moderate, heterogeneous tabular datasets and have demonstrated robust performance in previous studies of FRP-strengthened and FRP-reinforced concrete members [22,78].

The predictor set comprised 16 variables representing member geometry, concrete and internal reinforcement, FRP-system properties, and strengthening configuration. The geometric variables were beam depth *h*, web width *b_w_*, shear-span-to-effective-depth ratio *a*/*d*, and section type. Concrete and reinforcement were represented by concrete compressive strength *f_c_*′, longitudinal-reinforcement ratio *ρ_s_*, and the transverse-reinforcement index *ρ_v_f_yt_*. FRP-property variables comprised sheet rigidity *E_f_t_f_*, ultimate tensile strength *f_fu_*, strip continuity *w_f_*/*s_f_*, effective FRP-depth ratio *d_fv_*/*d*, and fiber angle *α*. Configuration variables represented anchorage, pre-cracking, and wrapping scheme. Categorical variables were binary encoded, with side bonding and rectangular sections as the respective reference categories. Full predictor definitions and encoding are provided in Section S3. For 72 specimens without transverse reinforcement, unreported fyt values were assigned the median value of 414 MPa; sensitivity analysis confirmed that this imputation had no influence because ρv = 0 for all such specimens (Section S3). Hyperparameters were selected by randomized search with internal five-fold cross-validation; the search space and selected values are reported in Table S3 and Table 1.

Model performance was evaluated using out-of-fold predictions, such that each specimen was predicted by a model that had not been trained on that observation. The baseline evaluation used shuffled ten-fold cross-validation with a fixed random seed of 42. Nested cross-validation was additionally used to verify that performance was not materially affected by hyperparameter tuning on the complete dataset; program grouping was preserved during grouped validation (Section S7).

Two program-controlled validation protocols were added. First, ten-fold GroupKFold validation assigned all specimens from a given experimental program to the same fold. Second, leave-one-program-out validation withheld each of the 43 represented programs in turn. To further examine transferability across programs, the leave-one-program-out error was decomposed into between-program bias, quantified by the mean residual of each held-out program, and within-program error. Within-program ordering was additionally assessed using program-centered observations and predictions and within-program Spearman correlations. Group ablation was repeated under both program-controlled validation protocols (Section S7). The random-fold protocol quantifies interpolation within the assembled database, whereas the grouped protocols provide a stricter assessment of transferability to an unseen experimental program. Model performance was evaluated using R^2^, RMSE, MAPE, and median absolute percentage error. Because percentage errors become unstable for small positive values of *V_f_*_,exp_, MAPE was also evaluated for the subset with *V_f_*_,exp_ > 30 kN. The response-surface model was developed to support interpretation and decision-making rather than to replace code-based design.

### 3.2. Triangulated Sensitivity and Attribution Framework

The fitted model was interpreted using five complementary methods: mutual information, SHAP values, permutation importance, group ablation, and Sobol′ variance decomposition. These methods were selected because they quantify different forms of feature relevance. No single method was interpreted in isolation.

#### 3.2.1. Mutual Information

Mutual information was used as a model-independent measure of marginal dependence between each predictor and *V_f_*_,exp_. Larger values indicate stronger, potentially nonlinear association with the response, but do not distinguish direct influence from association induced by correlated variables or program-level sampling.

#### 3.2.2. SHAP Attribution

SHAP values were calculated using exact TreeSHAP for the fitted XGBoost model. Global importance was quantified by the mean absolute SHAP value, and dependence plots were used to examine feature effects and interactions. SHAP feature-credit allocation can be unstable for correlated or redundant predictors [25,79].

#### 3.2.3. Permutation Importance

Permutation importance quantifies the dependence of the trained model on each predictor. Each predictor was permuted while the remaining variables were retained, and importance was measured from the resulting loss in predictive performance. Because the model is not retrained, permutation importance measures dependence of the fitted model rather than the unique information available after retraining.

#### 3.2.4. Group Ablation

Group ablation tested the unique predictive value of mechanically defined predictor groups. For each experiment, one group was removed, the response-surface model was retrained, and the complete ten-fold out-of-fold evaluation was repeated.

The ablation groups were as follows: *FRP properties* = {*E_f_t_f_*, *f_fu_*, *w_f_*/*s_f_*, *d_fv_*/*d*, *α*}, *transverse reinforcement* = {*ρ_v_f_yt_*}, *strengthening configuration* = {*Anch*, *WS_CW_*, *WS_UJ_*}, *member geometry* = {*h*, *b_w_*, *a*/*d*, *T*_sec_}, and *longitudinal reinforcement* = {*ρ_s_*}.

The ablation effect was defined as the reduction in out-of-fold R^2^ after removing each group.

#### 3.2.5. Sobol′ Variance Decomposition

Global variance-based sensitivity was evaluated using Sobol′ indices applied to the fitted XGBoost response surface. The first-order index represents the independent contribution of *X_i_* to response variance, whereas the total-order index includes all interactions involving *X_i_*. The difference *S_Ti_*-*S_i_* therefore quantifies the interaction contribution associated with that variable. Saltelli sampling was performed using a base sample size of 4096 (2^12^). Continuous variables were sampled over the 1st–99th percentile of their observed ranges, whereas categorical variables were sampled from their encoded categories defined in Section 3.1. Classical Sobol′ analysis assumes independent inputs. The resulting indices therefore characterize the fitted response surface under independent sampling rather than the empirical joint distribution of the experimental database.

#### 3.2.6. Interpretation Strategy

The methods were treated as complementary: mutual information quantified marginal association; SHAP allocated model output; permutation importance measured fitted-model dependence; group ablation assessed unique predictive information after retraining; and Sobol′ analysis quantified main and interaction contributions to response-surface variance. Disagreement was examined against the correlation structure, experimental sampling, and established shear mechanics. Only conclusions supported by several methods, or mechanically reconcilable where methods disagreed, were carried into the design discussion [25,79].

### 3.3. Uncertainty Analysis

Uncertainty in the material-efficiency comparisons was quantified using nonparametric bootstrap resampling. Break-even ratios were calculated as ratios of median material efficiencies. Each material subset was resampled independently with replacement 5000 times using a fixed random seed of 1, and the median-efficiency ratio was recalculated for each replicate. The 2.5th and 97.5th percentiles of the bootstrap distribution defined the 95% confidence interval. To reflect clustering of specimens within experimental programs, the principal CFRP:GFRP comparison was additionally evaluated using program-clustered bootstrap resampling. Measurement- and convention-related uncertainty in installed mass was then propagated by Monte Carlo simulation nested within the program-clustered bootstrap (5000 replicates, seed 1). In each replicate, a program-level factor represented the thickness convention and a material-level factor represented cured-composite density (1.45–1.75 g cm^−3^ for CFRP and 1.70–2.20 g cm^−3^ for GFRP). Two convention models were used. In the convention-informed model, reported thicknesses of 0.45 mm or less were treated as dry-fiber-equivalent values and converted to cured-laminate mass using a fiber volume fraction drawn uniformly between 0.30 and 0.60, while thicker products received a factor between 0.85 and 1.15. In the convention-agnostic stress test, every program received a log-uniform factor between 0.5 and 2.0 irrespective of the reported thickness (Section S6).

### 3.4. Robustness Analysis

Because the material groups differed in wrapping scheme, section type, anchorage, and other design characteristics, all between-material comparisons were interpreted as observational rather than causal. The CFRP:GFRP comparison was emphasized because it forms the basis of the proposed break-even framework. To assess robustness to structural composition, the principal material-efficiency ratio was recalculated within matched subsets comprising U-jacketed systems, rectangular sections, and unanchored specimens. A separate sensitivity analysis evaluated the exclusion of specimens with non-positive or undetermined *V_f_*_,exp_. Two variants were evaluated: all 17 excluded specimens, including the eight with undetermined values, were reintroduced as zero-efficiency observations; the nine measurable non-positive values were retained with their signed efficiencies, *V_f_*_,exp_/*m*, summarized by the ratio of signed medians, by a pooled ratio of total attributed contribution to total installed mass, and by a shifted-logarithm geometric-mean ratio (Section S11). The principal ratio was also recalculated within product-form strata: thin sheets and fabrics (reported *t_f_* ≤ 0.45 mm) and cured laminates and plates (*t_f_* > 0.45 mm).

Response-definition robustness was also examined by repeating the response-surface modeling using the size-normalized response *V_f_*_,exp_/(bwd) and log(*V_f_*_,exp_) as alternative targets. Permutation importance was additionally evaluated for the size-normalized response to assess whether the prominence of geometric variables was driven by first-order member-size scaling.

### 3.5. Subgroup Predictive Performance

Predictive performance was also evaluated within individual material, wrapping-scheme, anchorage, and section-type subsets using the baseline out-of-fold predictions. Subset-specific R^2^, RMSE, MAPE, and median absolute percentage error were calculated to identify regions of the database where predictive performance was reduced.

### 3.6. Design-Domain Applicability

The applicability domain of the decision maps was assessed using a kernel-density estimate constructed in the sheet-rigidity–continuity space. A 5% peak-density contour was adopted as the extrapolation boundary, while a higher-density contour identified the central data-supported region. Predictions outside the extrapolation boundary were identified as extrapolative, and the experimental observations were overlaid to distinguish interpolation from unsupported regions of the design space.

## 4. Results

### 4.1. Material-Efficiency Landscape

Material efficiency varied markedly across the assembled database. The 10th and 90th percentiles were 32 and 943 kN·m·kg^−1^, respectively, corresponding to an approximately 30-fold spread between the lower and upper deciles. This broad range indicates substantial variation in the shear contribution obtained per unit mass of installed FRP.

**Figure 1:**
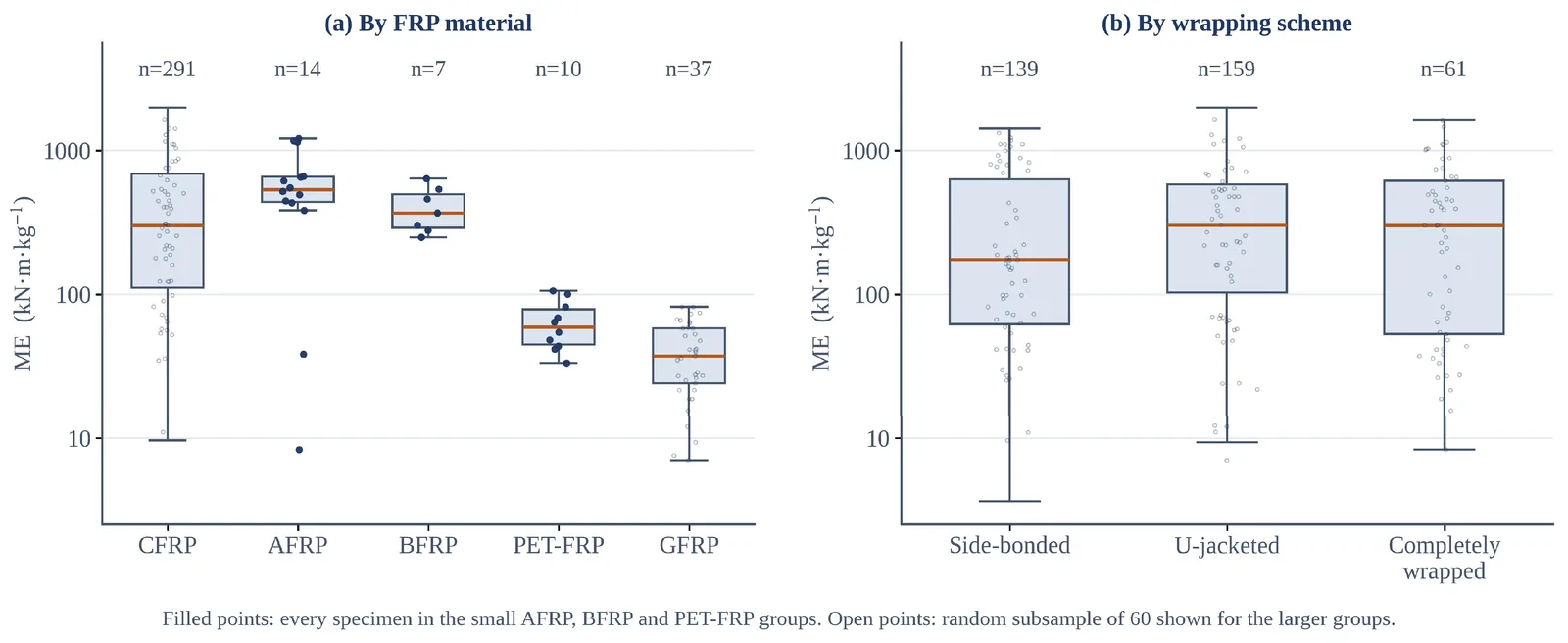
Material-efficiency distributions on a logarithmic scale: (**a**) by FRP material; (**b**) by wrapping scheme. Boxes show medians and interquartile ranges, and whiskers extend to 1.5 × IQR. Individual observations are overlaid, with subsampling used for larger groups to limit overplotting. Group sizes are annotated above each box.

Material-efficiency distributions are presented in Figure 1a, with the corresponding descriptive statistics summarized in Table 1. CFRP systems exhibited a median material efficiency of 303 kN·m·kg^−1^, compared with 38 kN·m·kg^−1^ for GFRP systems. AFRP had the highest observed median efficiency (538 kN·m·kg^−1^), whereas BFRP and PET-FRP exhibited medians of 370 and 59 kN·m·kg^−1^, respectively. The wide interquartile ranges indicate substantial within-material variability in material efficiency (Table 1). The distributions also exhibited considerable overlap, indicating that material type alone does not fully explain the observed variation in efficiency.

Wrapping configuration also influenced observed material efficiency. U-jacketed and completely wrapped systems exhibited comparable median values of 304 and 302 kN·m·kg^−1^, respectively, whereas side-bonded systems had a lower median of 175 kN·m·kg^−1^ (Figure 1b). The observed ordering is consistent with the bond-limited behavior of externally bonded shear strengthening. Side-bonded systems generally mobilize lower effective FRP strain because of premature debonding, whereas U-jackets and complete wraps provide greater bond development and confinement [14,17,80,81].

Anchored systems had a higher observed median efficiency than unanchored systems (376 versus 207 kN·m·kg^−1^). This contrast is mechanically plausible because anchorage delays debonding and increases the proportion of installed sheet capacity that can be mobilized [4,18,19]. However, anchorage was not assigned randomly across the database, and anchored systems differed from unanchored systems in member geometry, wrapping scheme, and strengthening intensity. The observed contrast was therefore interpreted as associative rather than causal.

### 4.2. Greenhouse-Gas and Cost Break-Even Rules

**Figure 2:**
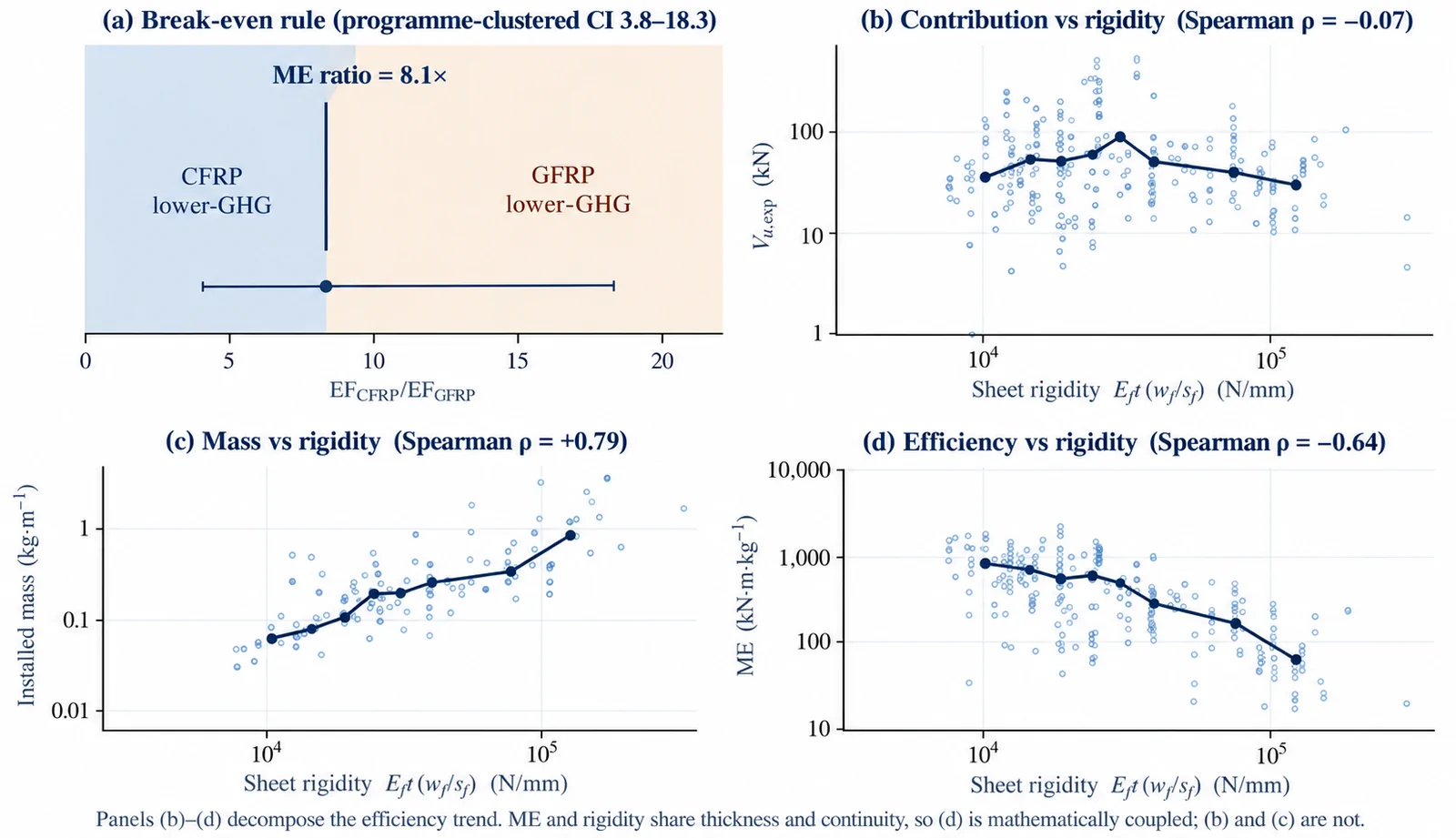
(**a**) Greenhouse-gas break-even rule for CFRP and GFRP: the emission-factor ratio EF*CFRP*/EF*GFRP* is compared with the observed median-efficiency ratio of 8.1 and its program-clustered 95% confidence interval of 3.8–18.3. (**b**–**d**) Attributed FRP contribution, installed sheet mass, and material efficiency plotted against installed sheet rigidity across the CFRP subset, with octile medians connected. Only panel (**d**) shares terms with the rigidity axis.

Table 2 and Figure 2a translate the observed material-efficiency contrasts into GHG break-even screening rules. The framework is presented for CFRP versus GFRP because GFRP represents the largest non-CFRP subset in the database (*n* = 37), providing a stronger empirical basis for comparison with CFRP (*n* = 291) than the smaller AFRP, BFRP, and PET-FRP subsets (*n* = 14, 7, and 10, respectively). The CFRP:AFRP and CFRP:PET-FRP median-efficiency ratios are therefore reported in Table 2 as indicative comparisons, whereas no break-even threshold is proposed for BFRP because of its limited sample size. The principal result was the observed CFRP:GFRP median-efficiency ratio of *ME_CFRP_*/*ME_GFRP_* = 8.1, with a program-clustered bootstrap 95% confidence interval of 3.8–18.3. When thickness-convention and density uncertainty were propagated together with program-level sampling, the 95% interval was 3.7–16.5 under the convention-informed model, and 2.8–21.3 under the convention-agnostic stress test; deterministic harmonization of thin-sheet thicknesses to cured-laminate mass gave point ratios of 7.0–7.9 (Table S6 and Table 1). The sampling interval therefore remains representative under plausible convention assumptions, whereas the wider stress-test interval provides a conservative decision band. This ratio should be interpreted as an observational, database-level, sheet-only screening benchmark rather than as a universal material-selection rule: it describes an association within the present database, not a causal material effect, and its use as a fixed design threshold would be premature.

This ratio defines the sheet-level cradle-to-gate GHG break-even threshold between the two materials. GFRP becomes the lower-GHG option per unit of delivered shear contribution only when *EF*_CFRP_/*EF*_GFRP_ > 8.1. If the project-specific CFRP:GFRP emission-factor ratio is below 8.1, CFRP remains the lower-GHG option because, at the observed efficiency ratio, its greater median contribution per unit mass offsets its higher per-kilogram greenhouse-gas burden.

Because the material subsets differed in wrapping scheme, section type, and anchorage, the overall ratio was treated as an observational database contrast rather than a causal material effect. Nevertheless, the contrast persisted within matched structural subsets (Table S9 and Table 1). The CFRP:GFRP ratio was 9.33 for U-jacketed systems, 10.92 for rectangular sections, and 7.54 for unanchored systems. Within product-form strata, the ratio was 9.9 (program-clustered 95% CI: 5.0–21.7) for thin sheets and fabrics and 4.8 (1.9–21.7) for cured laminates and plates, so CFRP retained the higher median efficiency in every stratum examined (Table S9 and Table 1). These results indicate that the overall contrast was not driven solely by one dominant configuration variable or by the thickness convention. However, the observed CFRP–GFRP efficiency contrast should not be interpreted as a causal material effect, because differences in other program- and specimen-level characteristics may remain even within the matched structural subsets.

The exclusion of non-positive contributions had only a modest effect on the principal ratio. Reintroducing all 17 excluded specimens as zero-efficiency observations reduced the ratio from 8.07 to 7.37. Retaining the nine measurable non-positive values with their signed efficiencies gave a ratio of 7.51 (program-clustered 95% CI: 3.6–17.8); the pooled ratio of total attributed contribution to total installed mass was 7.88 (4.0–16.9), and the back-transformed shifted-logarithm geometric-mean ratio was 8.04 (5.0–15.7) (Table S11 and Table 2). The break-even threshold is therefore not an artifact of discarding adverse or negligible FRP contributions.

The confidence interval defines the uncertainty surrounding the break-even threshold. Project-specific emission-factor ratios substantially below the lower confidence bound support a comparatively robust CFRP preference, whereas ratios substantially above the upper confidence bound support GFRP. For conservative screening, the bounds of the stress-test interval (2.8 and 21.3) should be used in place of the sampling interval. Ratios within the interval should not be interpreted as indicating a decisive environmental advantage. In such cases, secondary considerations—including durability, constructability, installation time, local availability, fire performance, maintenance requirements, and project-specific constraints—should contribute to material selection. The same material-efficiency ratio may also be used as a first-order cost-screening metric. Under this interpretation, GFRP becomes more material-efficient per unit cost than CFRP only when *P_CFRP_*/*P_GFRP_* > 8.1, provided that the comparison refers to equivalent cured-composite products on the same quantity basis. The CFRP:AFRP median-efficiency ratio was 0.56 (95% confidence interval: 0.38–0.73), indicating higher observed median efficiency for AFRP within the available dataset; however, this comparison is based on only 14 AFRP specimens and should not be interpreted as evidence of a general material advantage. The CFRP:PET-FRP median-efficiency ratio was 5.1 (95% confidence interval: 3.3–7.2), although this comparison is likewise constrained by the limited sample size.

Figure 2b–d decompose the relationship between installed sheet rigidity and material efficiency across the CFRP subset. The rigidity–efficiency relationship and its material-composition dependence are examined quantitatively in Section 4.4.

### 4.3. Predictive Performance and Interpretation of the Attributed FRP Contribution

**Figure 3:**
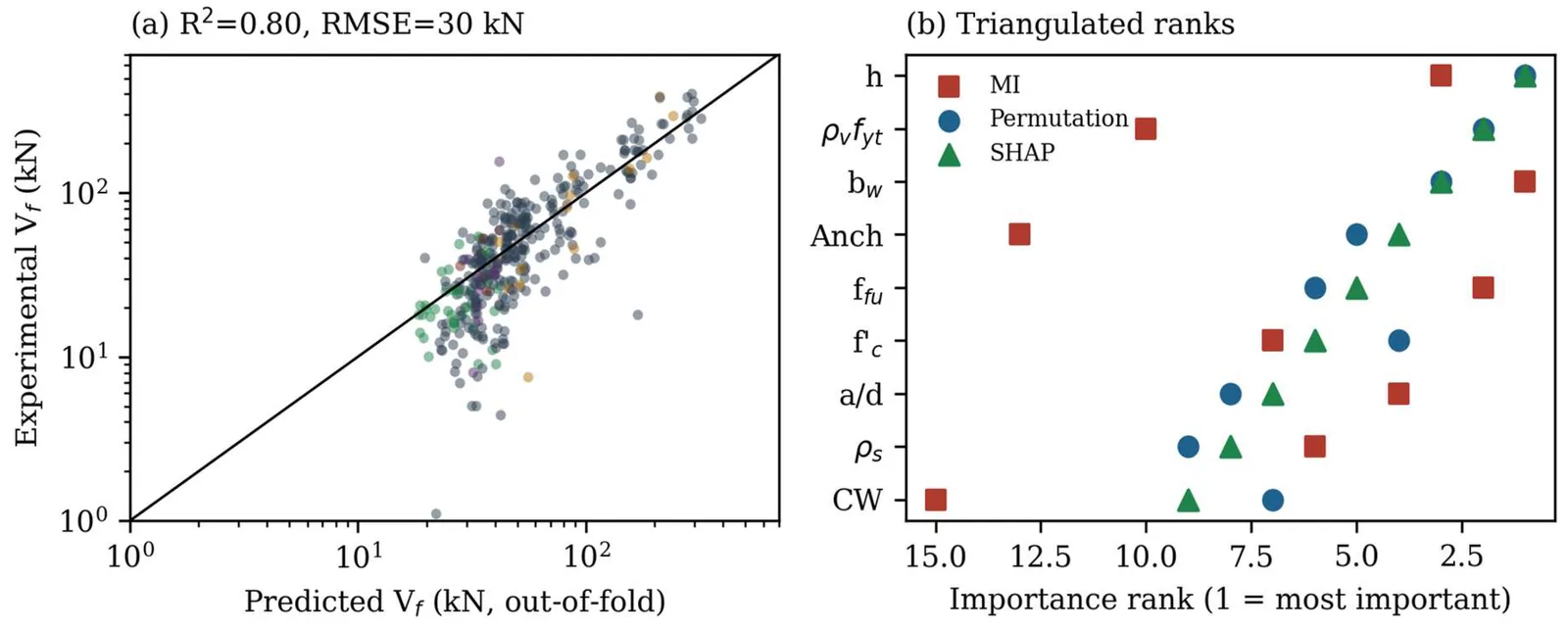
(**a**) Out-of-fold prediction of the experimentally attributed FRP shear contribution; both axes are logarithmic. All observed and predicted values are positive. (**b**) Feature rankings obtained using mutual information, permutation importance, and SHAP, highlighting the differences between marginal and conditional attribution. The different dot colors in panel (**a**) distinguish specimens from different experimental programmes and do not represent different FRP material types.

The XGBoost model achieved a random ten-fold out-of-fold coefficient of determination of R^2^ = 0.802, with an RMSE of 30.4 kN and a mean absolute error (MAE) of 20.3 kN (Figure 3a). The overall mean absolute percentage error (MAPE) was 62.6%, whereas the median absolute percentage error was 27.4%. Restricting the analysis to the 232 specimens with V_*f*,exp_ > 30 kN reduced the MAPE to 27.0%, indicating that the large overall value is primarily driven by specimens with very small attributed FRP contributions.

Prediction errors remain substantial relative to the mean attributed FRP contribution (RMSE ≈ 47% of the mean *V_f_*_,exp_ of 64 kN), partly because *V_f_*_,exp_ is obtained as the attributed difference between two experimentally measured capacities and therefore incorporates variability associated with both measurements, a characteristic widely recognized for FRP shear-contribution models [82]. Accordingly, the model is intended for attribution, interpretation, and efficiency mapping rather than direct design calculations.

Predictive performance decreased substantially when the validation protocol prevented information sharing between experimental programs. Under program-grouped ten-fold cross-validation, the coefficient of determination decreased to R^2^ = 0.526 with an RMSE of 47.0 kN, while leave-one-program-out validation further reduced performance to R^2^ = 0.394 with an RMSE of 53.1 kN (Table S7 and Table 1). This decline indicates that a portion of the predictive skill observed under random cross-validation reflected interpolation within experimental families rather than transfer to previously unseen experimental programs. Because specimens from the same program share materials, geometry, detailing, and testing protocols, random splitting can place program-specific characteristics in both training and test folds.

The loss of transferability has a specific structure. Differences between program means account for 74% of the variance of *V_f_*_,exp_ in the database. Under leave-one-program-out validation, 64% of the mean squared error arose from a program-level offset, the mean residual of the held-out program, compared with 12% under random cross-validation (Table S7 and Table 2). The model therefore predicts the level of an unseen program poorly, and program means were reproduced with R^2^ = 0.52 only, because that level reflects conditions that the 16 predictors do not describe, such as the companion-control capacity, material batch, fabrication quality, loading arrangement, and the convention used to report sheet thickness. Within programs, predictions retained moderate ordering skill: the median Spearman correlation between observed and predicted contributions within held-out programs was 0.45 and was positive in 32 of the 40 programs with at least four specimens. The negative R^2^ values obtained for 33 of the 43 individual programs (Section S7) should be read in this light, because each is referenced to a within-program variance that is often small, so a moderate level offset yields a negative value even when the ordering of specimens is retained. The response surface is therefore better suited to comparing alternatives on a given member, where a common offset largely cancels, than to predicting the absolute contribution of a new program.

The attribution conclusions were re-examined under the same program-controlled protocols (Table S7 and Table 3). With program-grouped validation, removing member geometry reduced R^2^ by 0.43 and removing transverse reinforcement by 0.13, whereas removing the FRP-property group did not reduce R^2^ (Δ*R*^2^ = −0.04; −0.09 under leave-one-program-out validation). Across the 43 leave-one-program-out models, varying beam depth over its 10th–90th-percentile range Figure 6 at the reference configuration described in Section 4.4 changed the predicted contribution by a median of 51 kN, compared with 0.3 kN for sheet rigidity and 2.3 kN for strip continuity, and the depth effect exceeded both sheet-property effects in every model. The member-dominated attribution and the near-invariance of the predicted contribution to sheet rigidity and continuity (Section 4.4) are therefore not artifacts of random splitting; sheet-property effects learned under random splitting appear, if anything, partly program-specific. The absolute values and fine structure of the maps, by contrast, remain conditional on the densely sampled CFRP region.

#### 4.3.1. Comparison of Attribution Methods

**Figure 4:**
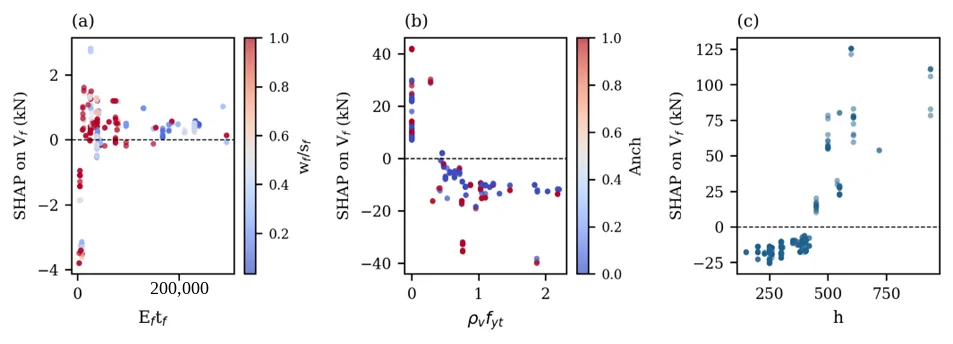
SHAP dependence of the predicted FRP contribution on: (**a**) sheet rigidity, colored by strip continuity; (**b**) stirrup index, colored by anchorage; and (**c**) beam depth.

The attribution methods produced both consistent and divergent rankings. Beam depth *h*, stirrup index *ρ_v_f_yt_*, and web width *b_w_* were consistently prominent under permutation importance and SHAP. Mutual information, however, ranked FRP tensile strength first, followed by beam depth and web width, while placing the stirrup index much lower. This disagreement reflects the difference between marginal association and conditional predictive dependence. FRP tensile strength and other sheet properties were associated with *V_f_*_,exp_ across the database, potentially reflecting their co-occurrence with particular experimental programs and member configurations. Once member geometry, internal reinforcement, and configuration were included simultaneously, however, their additional predictive information was substantially smaller [25,83]. The eight highest-ranked features are reported in Table 3 and illustrated in Figure 3b. Figure 4 shows the corresponding SHAP dependence relationships for sheet rigidity, the stirrup index, and beam depth, including an anchorage-dependent pattern for the stirrup index.

#### 4.3.2. Group Ablation Importance

The baseline random-fold out-of-fold *R*^2^ was 0.802; the complete ablation results are reported in Table S5 and Table 1. Removing the member-geometry group reduced *R*^2^ to 0.715, corresponding to Δ*R*^2^ = 0.087. Removing the transverse-reinforcement group reduced *R*^2^ to 0.761, Δ*R*^2^ = 0.040. Removing the strengthening-configuration group reduced *R*^2^ to 0.779, Δ*R*^2^ = 0.022. By contrast, removing the FRP-properties group—sheet rigidity, tensile strength, continuity, effective-depth ratio, and fiber angle—reduced *R*^2^ only to 0.799, Δ*R*^2^ = 0.002. Longitudinal reinforcement produced the same ΔR^2^ = 0.002. Thus, FRP properties and longitudinal reinforcement contained little unique predictive information once the remaining groups were retained, whereas member geometry and transverse reinforcement were substantially less replaceable by the remaining predictors, consistent with earlier studies [41,84].

#### 4.3.3. Sobol′ Decomposition

The Sobol′ analysis supported the same member-dominated trend. Beam depth and stirrup index had first-order Sobol′ indices (*S_i_*) of 0.311 and 0.317, respectively, and total-order indices ($S_{T_{i}}$) of 0.485 and 0.431. Web width and concrete compressive strength had lower $S_{T_{i}}$ values of 0.090 and 0.089. Anchorage had $S_{T_{i}}$ = 0.048. The principal FRP-system variables each had *S_T_* values close to or below 0.01 (Table S4 and Table 1). The summed *S_i_* values were approximately 0.821, indicating that about 18% of the fitted-response variance under the independent-input Sobol′ sampling was associated with interaction effects.

**Figure 5:**
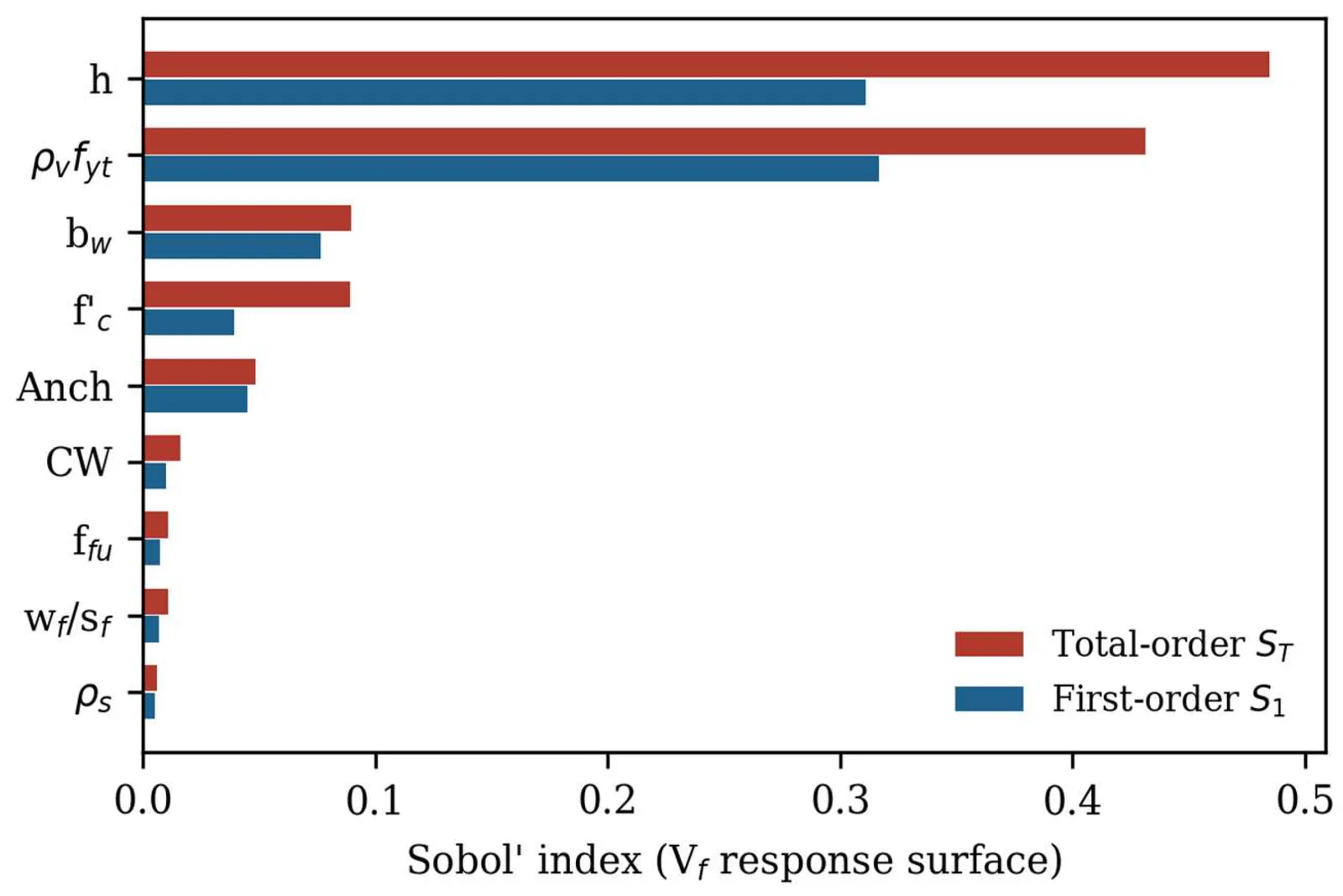
Sobol′ first-order and total-order indices of the fitted *V_f_*_,exp_ response surface. The difference between the total- and first-order indices represents interaction effects.

The substantial gaps between *S_i_* and $S_{T_{i}}$ for beam depth and stirrup index indicate that their importance arose not only from independent effects but also from interactions with other variables (Figure 5). This interaction structure is consistent with the member- and bond-dependent mechanics of FRP shear strengthening [16,17,41,84].

#### 4.3.4. Target-Normalization Robustness

The primary model used the absolute FRP contribution in kilonewtons. Because larger members naturally permit larger absolute contributions, geometric variables may partly act as size proxies. The response-surface modeling was therefore repeated using *V_f_*_,exp_/*b_w_d* and log(*V_f_*_,exp_) as alternative targets.

For the size-normalized target, out-of-fold *R*^2^ decreased to 0.520 (Table S8 and Table 1). The stirrup index became the leading permutation variable, followed by longitudinal reinforcement, anchorage, *a*/*d*, beam depth, and *d_fv_*/*d*. Beam depth therefore lost its dominant rank after first-order size scaling was removed, confirming that part of its prominence in the absolute model reflected member size. Nevertheless, member, reinforcement, and configuration variables remained prominent relative to the principal sheet-property variables. The member-conditioned interpretation was therefore not solely an artefact of expressing *V_f_*_,exp_ in absolute units.

#### 4.3.5. Predictive Performance Across FRP Systems and Configurations

Predictive performance also varied strongly across subsets. The CFRP subset achieved an out-of-fold *R*^2^ of 0.806, whereas GFRP, BFRP, and PET-FRP exhibited negative or near-zero values. AFRP achieved *R*^2^ = 0.731, although this estimate is based on only 14 specimens. The U-jacketed, side-bonded, and completely wrapped subsets achieved *R*^2^ values of 0.817, 0.774, and 0.743, respectively. Anchored systems achieved the highest subset performance, *R*^2^ = 0.848, whereas unanchored systems achieved *R*^2^ = 0.690. The resulting efficiency maps should therefore be interpreted primarily as a description of the densely represented CFRP-like region rather than as a universal predictor for all fiber systems.

### 4.4. Diminishing Material Returns and Efficiency Maps

Material efficiency generally decreased with increasing installed sheet rigidity across the CFRP subset. Across the rigidity octiles in Figure 2d, the octile median decreased from 707 to 41 kN·m·kg^−1^ as *E_f_t_f_*(*w_f_*/*s_f_*) increased approximately twelvefold, from 10,340 to 121,297 N/mm, corresponding to a seventeen-fold reduction in efficiency (specimen-level Spearman ρ = −0.64). This decline primarily reflected increasing installed material rather than increasing attributed shear contribution. Across the same range, the median *V_f_*_,exp_ changed only modestly, from 36.8 to 31.2 kN (Figure 2b; ρ = −0.07), whereas the installed mass increased approximately fifteenfold, from 0.056 to 0.819 kg·m^−1^. Because installed rigidity and installed mass share sheet thickness and strip continuity, part of the inverse relationship is a mathematical consequence of the material-efficiency definition. Nevertheless, the attributed shear contribution remained nearly constant while installed material increased by approximately an order of magnitude. This behavior is consistent with, although it does not uniquely demonstrate, bond-limited FRP mechanics, whereby increasing sheet thickness, stiffness, or continuity beyond the mobilizable bond response may add material without a proportional gain in shear contribution [14,15,80].

**Figure 6:**
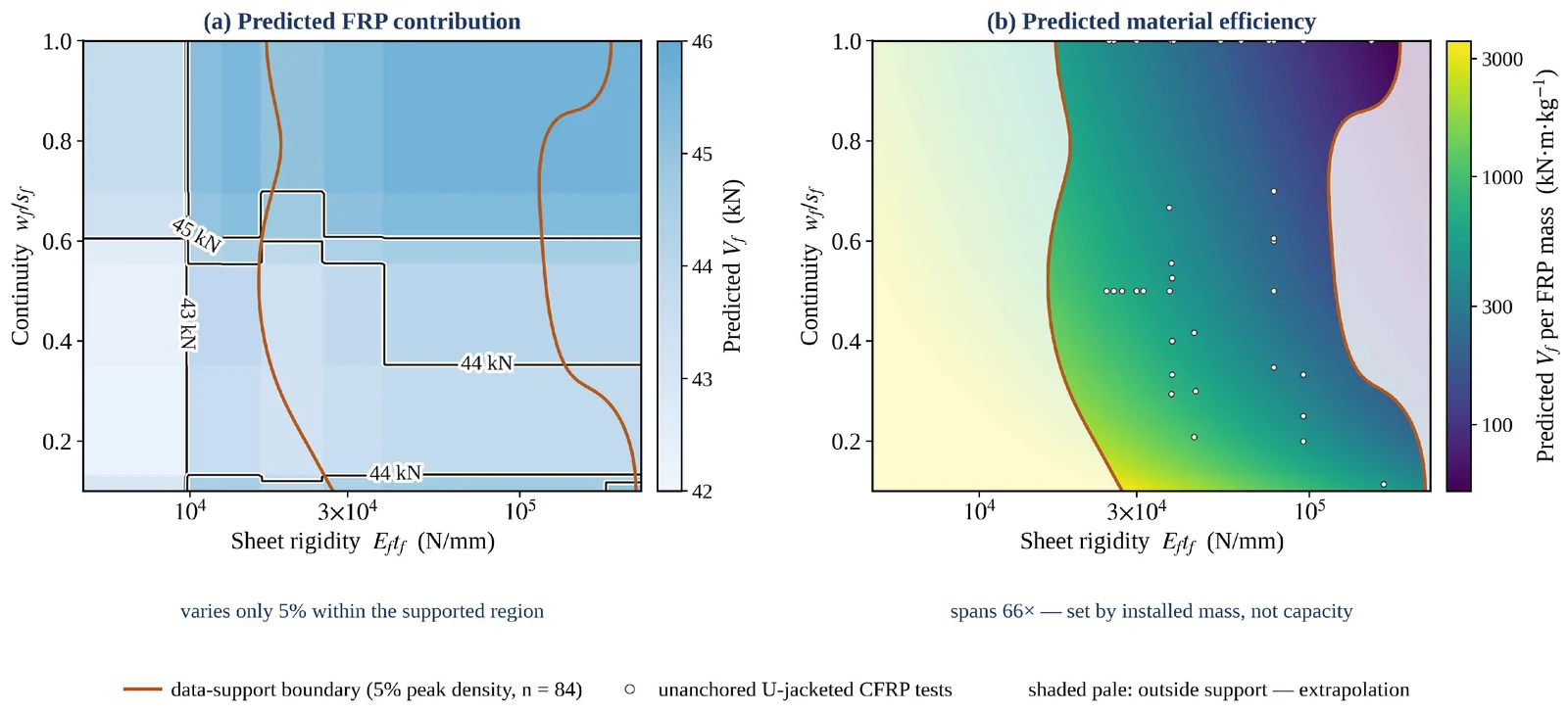
Predicted (**a**) attributed FRP shear contribution, with iso-contours in kN; (**b**) material efficiency in kN·m·kg^−1^ on a logarithmic scale, over the sheet-rigidity–continuity plane for a representative unanchored U-jacketed CFRP beam. Orange line: 5% data-support boundary computed from the 84 tests of this configuration, with tests overlaid in (**b**); pale regions lie outside that boundary and are extrapolative.

Figure 6 shows the predicted FRP contribution (a) and the corresponding material efficiency (b) over the sheet-rigidity–continuity plane, with all remaining inputs held at the medians of the 84 unanchored U-jacketed CFRP specimens (h = 390 mm, b*w* = 152 mm, a/d = 3.0, f′*c* = 32 MPa, T-section). The predicted contribution is nearly invariant across the plane, spanning only 43.3 to 45.6 kN within the data-supported region, approximately 5%. By comparison, varying beam height alone from 200 to 610 mm changes the predicted contribution from 38.5 to 128.3 kN, a factor of 3.3. Installed mass, by contrast, rises 68-fold across the same region, from 0.013 to 0.848 kg·m^−1^. Predicted efficiency therefore spans 54 to 3537 kN·m·kg^−1^, a factor of 66, and is greatest where the installation is lightest. Regions beyond the 5% support boundary are shown pale and are not read quantitatively.

The highest predicted efficiencies occur in the low-rigidity, low-continuity corner. However, this region is sparsely represented in the database and may correspond to widely spaced strips with uncertain bond reliability. It is therefore not interpreted as a recommended design region. Accordingly, the decision map should be applied only within the data-supported region and along the required code-compliant contribution contour.

The practical implication is not that rigidity or continuity should be minimized. Rather, once the required contribution, anchorage, effective strain, and failure mode have been established through code-based design, excessively rigid or overdense sheet layouts may consume substantially more composite without delivering proportional structural benefit. The framework therefore identifies lower-material-intensity alternatives that satisfy the required structural contribution while remaining within the supported design domain.

### 4.5. Benchmarking Against Published Predictive Models

The baseline random-fold performance obtained in this study was within the range reported for FRP-shear machine-learning results. Previous ensemble-tree and constrained-neural-network studies have reported coefficients of determination of approximately 0.80–0.96 for total shear capacity or closely related targets [22,23,77,78,82]. Direct comparison, however, requires caution because the present target was the experimentally attributed FRP contribution, *V_f_*_,exp_ = *V*_strengthened_ − *V*_control_ rather than total shear capacity. The differenced response contains variability from both the strengthened and control specimens and is consequently more difficult to predict than total capacity. The random-fold value of *R*^2^ = 0.80 is therefore within the range of published performance despite the more demanding response definition.

Program-grouped validation reduced *R*^2^ to 0.53, and leave-one-program-out validation reduced it to 0.39. SHAP rankings computed on random-split models may reflect program-specific associations as well as general structural relationships. The decision maps were not presented as validated predictors for unseen experimental programs, and extrapolative regions were identified explicitly. The program-grouped ablation reported in Section 4.3 nevertheless indicates that the group-level ranking of member, reinforcement, and FRP-property effects was preserved when entire programs are withheld.

Closed-form models based on effective FRP strain and bond-limited mechanics remain essential for design because they provide transparent safety checks and code compatibility [14,41,80]. Their accuracy, however, is limited by the difficulty of representing the transition between debonding and rupture across different member and strengthening configurations. Response-surface models can capture this heterogeneity more flexibly but require strict control of leakage, extrapolation, and interpretability.

The principal contribution of the proposed framework is therefore not a uniquely high predictive score. It is a transparent evaluation framework that distinguishes interpolation from transfer to unseen programs, identifies extrapolative regions and subgroup limitations, separates marginal association from unique predictive information, and links structural response to material-efficiency-based decision support.

## 5. Discussion

The results suggest that the material efficiency of externally bonded FRP shear strengthening appears to be governed by the interaction between the FRP system and the strengthened member rather than by sheet properties alone. The approximately 30-fold spread in ME and the greater importance of geometry and transverse reinforcement indicate that efficient polymer-composite utilization depends on how effectively the strengthened member mobilizes the installed FRP.

The efficiency maps should therefore be interpreted as empirical, member-conditioned surfaces rather than material-property maps. Their tendency to favor relatively light, low-rigidity systems is consistent with finite effective bond length and limited strain mobilization before debonding [14,15,80], although the database cannot isolate bond limitation from correlated design variables.

The sparsely populated high-efficiency region at very low rigidity and continuity should not be interpreted as a design recommendation; the density boundary identifies the better-supported interpolation region.

### 5.1. Mechanical Interpretation and Implications for Retrofit Selection

The member-dominated attribution is mechanically plausible because FRP mobilization depends on diagonal-crack development, which is governed by section geometry, shear-span-to-depth ratio, concrete properties, and internal transverse reinforcement. Consequently, nominally identical FRP systems can develop substantially different contributions on different members [16,41,84].

Interaction with internal stirrups is particularly important because FRP and transverse steel resist the same crack system. Previous studies have demonstrated FRP–steel interaction and changes in stirrup strain at failure [16,17,41,84]. Accordingly, V_*f*,exp_ should be interpreted as a system-level increment under the tested reinforcement condition, not as an intrinsic property of the composite sheet. This interpretation is consistent with the prominence of the stirrup index across the attribution analyses. Beam depth was likewise influential for the absolute target, although its dominance weakened after section-size normalization.

At lower a/d ratios, arching action and concrete-cover separation can reduce the effectiveness of externally bonded FRP as behavior shifts from conventional diagonal-tension response [85,86]. The framework should therefore not be extrapolated to deep or strongly arch-dominated members without appropriate structural analysis.

By delaying debonding, anchorage can increase the fraction of installed composite capacity that is mobilized [4,18,19]. The higher median ME of anchored systems is consistent with this mechanism. However, the observational database contrast should not be interpreted causally because anchored and unanchored specimens were not experimentally matched.

Similarly, the results do not imply unconstrained minimization of sheet rigidity or continuity. Rather, among alternatives providing the required code-compliant contribution, lower-material-intensity systems may be preferable provided that bond, anchorage, durability, and constructability requirements are satisfied.

### 5.2. Multi-Method Attribution and Mechanical Interpretation

The attribution methods provided complementary views of the fitted response. Mutual information identified strong marginal associations, including FRP tensile strength, whereas SHAP and permutation importance emphasized beam depth, stirrup index, and web width within the multivariable model. Because correlated predictors can redistribute feature importance [25,79], group ablation was used to assess unique predictive information. Removing geometry or transverse reinforcement caused substantially larger losses in out-of-fold *R*^2^ than removing the FRP-system block.

Sobol′ analysis supported this interpretation. Beam depth and stirrup index showed the largest total-order effects, while individual FRP-system variables contributed less to global fitted-response variance. The approximately 18% interaction share further indicates that the response cannot be represented by isolated main effects alone. Thus, the relatively high marginal importance but low ablation importance of some FRP properties is not contradictory. Their predictive contribution is strongly conditioned by member geometry, reinforcement, configuration, and experimental context. Across methods, the stable conclusion is that member geometry and transverse reinforcement carry greater predictive and variance-based importance for *V_f_*_,exp_ than isolated FRP-sheet properties.

### 5.3. Relationship to Code-Based FRP Design

The framework complements rather than replaces established FRP shear-design procedures. Required strengthening contribution, effective strain, debonding limit, wrapping configuration, anchorage, and safety factors must still satisfy the applicable design provisions, including ACI PRC-440.2 [87], fib guidance [88], CNR-DT 200 [89], Concrete Society TR55 [90], or equivalent standards.

**Figure 7:**
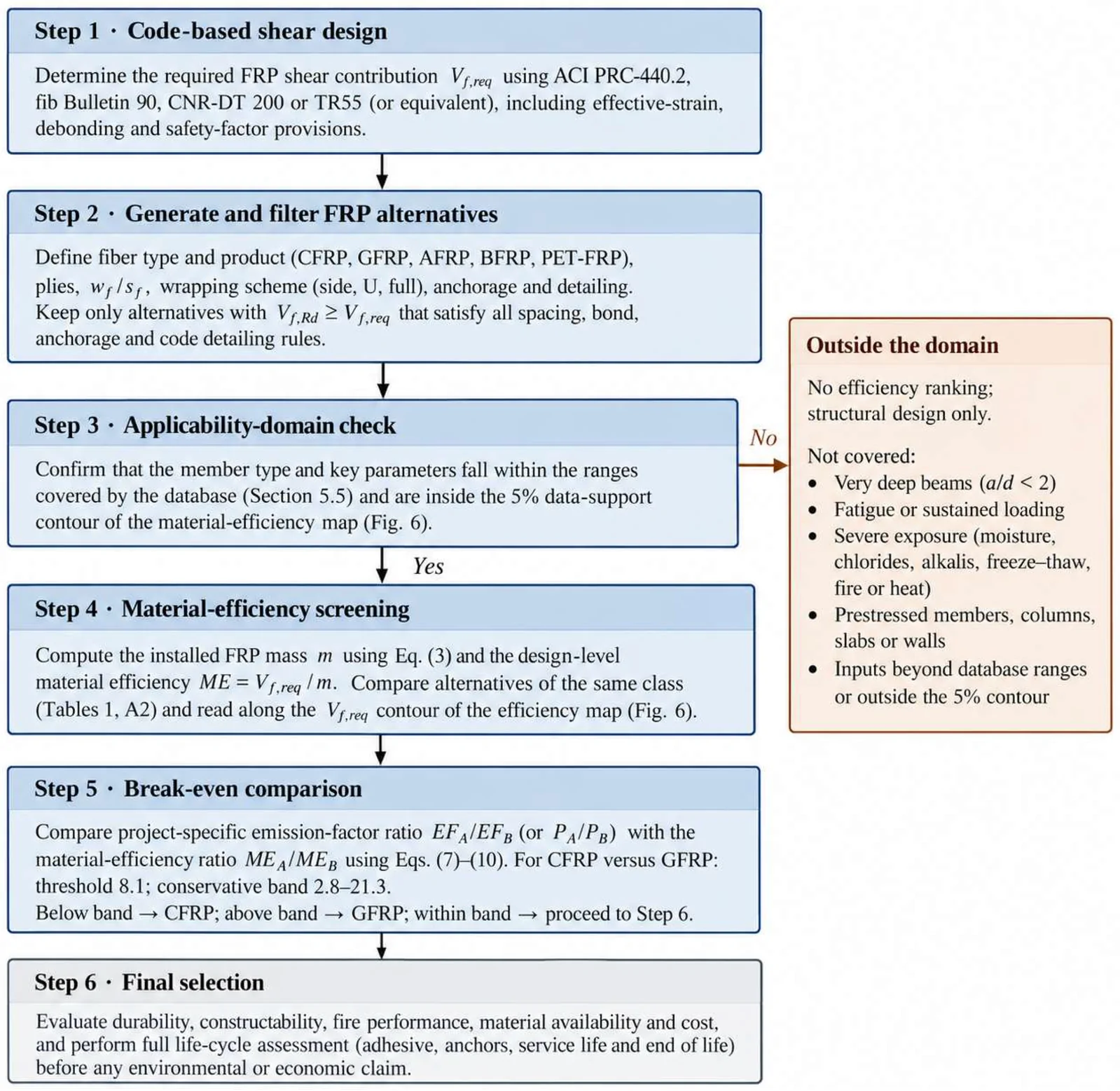
Procedure linking code-based FRP shear design with the material-efficiency and break-even screening proposed in this study. The applicability-domain check (Step 3) precedes the efficiency comparison, so that extrapolative alternatives and members outside the scope of the database are excluded from the ranking.

Material-efficiency and break-even analyses should therefore follow structural design; the complete procedure is summarized in Figure 7. In Step 1, shear design according to the governing provisions yields the required FRP contribution, *V_f_*_,req_, including the effective-strain, debonding and safety-factor requirements. In Step 2, candidate systems (fiber type and product, number of plies, strip width and spacing, wrapping scheme, and anchorage) are generated, and only those that provide *V_f_*_,req_ and satisfy all spacing, bond, anchorage and detailing rules are retained. In Step 3, each admissible alternative is checked against the applicability domain: alternatives outside the database ranges listed in Section 5.5, or outside the 5% data-support contour of the efficiency map, are removed from the efficiency comparison and assessed by conventional structural means only. Placing this check before the comparison ensures that no extrapolative option influences the ranking. In Step 4, the installed mass of each remaining alternative is calculated from Equation (3). Because every alternative delivers *V_f_*_,req_, its design-level efficiency is *V_f_*_,req_/*m*; this value is compared with the efficiencies achieved by tested systems of the same class (Table 1 and Table A2) and read along the *V_f_*_,req_ contour of the efficiency map, which identifies alternatives whose code-predicted contribution would imply an efficiency well beyond the tested range. In Step 5, the project-specific emission-factor or price ratio is compared with the break-even threshold, Equations (7)–(10); for CFRP and GFRP, the threshold is 8.1, and the conservative band is 2.8–21.3. In Step 6, ratios within the band, and the final choice in every case, are resolved with secondary criteria and, where a life-cycle claim is intended, with a full LCA.

The framework thus supports selection rather than sizing, and the efficiency map should be interpreted along the required-contribution contour established by structural design. A mathematically efficient point may still violate minimum reinforcement, spacing, bond, anchorage, durability, or constructability requirements. The relevant objective is therefore comparison among structurally admissible alternatives rather than unconstrained maximization of ME.

### 5.4. Greenhouse-Gas Interpretation and Life-Cycle Boundary

The break-even formulation deliberately separates database-derived structural performance from project-specific environmental inventories because FRP emission factors vary with product form, precursor route, electricity mix, resin content, allocation assumptions, and system boundary [12,29,30,31]. The CFRP:GFRP efficiency ratio is therefore not an environmental ranking; it is a structural threshold to be compared with a boundary-compatible project-specific emission-factor ratio. The same logic applies to cost when equivalent product and quantity bases are used.

The adopted boundary is cradle-to-gate production of the cured composite sheet. Adhesive, primer, anchors, transport, site energy, labor, maintenance, and end-of-life treatment are excluded (Table S12 and Table 1). The resulting criterion therefore represents a sheet-level GHG break-even threshold, not total life-cycle emissions nor total installed retrofit cost. Environmental factors must consequently refer to equivalent product forms and system boundaries; for example, a dry carbon-fiber factor should not be compared directly with that of a cured GFRP sheet or a fully installed strengthening system.

Broader life-cycle boundaries could alter the ranking. Adhesive demand scales with bonded area rather than with composite mass, while end-of-life recovery [30,31], durability, and replacement requirements may favor either system. These effects require project-specific life-cycle data and are outside the present comparison.

The direction of the most important boundary extension can nevertheless be bounded from the database. Adhesive, saturant and primer demand scale primarily with bonded area rather than with composite mass. Let *q* denote the cradle-to-gate greenhouse-gas emission of these consumables per square meter of bonded area, *A_i_* = (*w_f_*/*s_f_*)*p* the bonded area per meter of beam, and *AE_i_* = *V_f_*_,exp_/*A_i_* the attributed contribution per unit bonded area. Including this term, GFRP is the lower-GHG option only when

(11) $$\frac{{\mathrm{EF}}_{\mathrm{CFRP}}}{{\mathrm{EF}}_{\mathrm{GFRP}}}>\frac{{\mathrm{ME}}_{\mathrm{CFRP}}}{{\mathrm{ME}}_{\mathrm{GFRP}}}+\frac{q{\mathrm{ME}}_{\mathrm{CFRP}}}{{\mathrm{EF}}_{\mathrm{GFRP}}}\left(\frac{1}{{\mathrm{AE}}_{\mathrm{GFRP}}}-\frac{1}{{\mathrm{AE}}_{\mathrm{CFRP}}}\right)$$

In the database, the median *AE* was 159 kN·m^−1^ for CFRP and 53 kN·m^−1^ for GFRP, a ratio of 3.0 (program-clustered 95% CI: 2.1–4.7; Table S12 and Table 2). Because *AE*_GFRP_ < *AE*_CFRP_, the additional term in Equation (11) is positive: Including area-dependent consumables shifts the break-even threshold in favor of CFRP relative to the sheet-only criterion; a sheet-level GFRP preference should consequently be confirmed using project-specific adhesive data. Service life acts multiplicatively. If system *i* requires *N_i_* installations over the reference period, the threshold becomes

(12) $$\frac{{\mathrm{EF}}_{\mathrm{CFRP}}}{{\mathrm{EF}}_{\mathrm{GFRP}}}>\frac{{\mathrm{ME}}_{\mathrm{CFRP}}}{{\mathrm{ME}}_{\mathrm{GFRP}}}\cdot \frac{N_{\mathrm{GFRP}}}{N_{\mathrm{CFRP}}}$$

so a shorter replacement interval for either system shifts the threshold in direct proportion. End-of-life burdens per kilogram can be added to *EF_i_* without changing the form of Equation (8). Because relative durability, replacement frequency, anchorage demand and end-of-life routes are project-specific and were not reported by the source programs, the criterion is presented as a sheet-level screening threshold and should not be interpreted as a life-cycle environmental preference; a full LCA that includes the excluded stages is required before such a claim is made.

### 5.5. Limitations and Scope of Application

The analysis is a secondary synthesis of published tests, and its results are indicative rather than directly applicable as design values. Several limitations define the intended use of the framework. *V_f_*_,exp_ is obtained by differencing strengthened and control capacities and inherits uncertainty from both measurements. The database is CFRP-dominated (291 of 359 specimens); GFRP, AFRP, BFRP, and PET-FRP are represented by 37, 14, 7, and 10 specimens from seven, two, one, and one programs, respectively, so comparisons involving these materials rest on few programs and products and cannot be generalized beyond them. The CFRP:GFRP comparison remains observational despite matched-stratum analyses, and residual confounding from program-specific details and specimen configuration cannot be excluded. Nominal cured-composite densities were required because specimen-specific densities and fiber-volume fractions were generally unavailable; fiber areal weight, weave architecture, and manufacturing route could not be controlled individually, and their effects are represented only through the reported sheet properties, the within-material scatter, and the program-clustered and convention-propagated intervals. The environmental comparison is restricted to cradle-to-gate cured-sheet production. Sobol′ indices describe the fitted response surface under independently sampled inputs rather than the empirical correlated population. Program-controlled validation also showed limited transferability beyond the assembled evidence base, indicating that the efficiency maps should be interpreted primarily within the densely sampled CFRP-dominated region. Subset-specific performance was unreliable for the small GFRP, BFRP, and PET-FRP datasets (Table S10 and Table 1). Finally, the framework applies within the ranges covered by the database: beam depth 150–940 mm, web width 100–600 mm, *a*/*d* between 2.0 and 3.9, concrete compressive strength 12.7–60 MPa, and rectangular or T-sections with side-bonded, U-jacketed, or completely wrapped FRP. Deep beams (*a*/*d* < 2, represented by only 33 specimens), members under sustained or fatigue loading, members exposed to severe environments (moisture, chlorides, alkalis, freeze–thaw, elevated temperature, or fire), prestressed members, columns, slabs, walls, and long-term bond durability are outside its scope (Figure 7).

## 6. Conclusions

This study developed a literature-based material-efficiency framework for externally bonded FRP shear strengthening using a database of 359 published tests from 43 experimental programs. The main conclusions are as follows:

1. Material efficiency varied approximately 30-fold, from 32 kN·m·kg^−1^ at the 10th percentile to 943 kN·m·kg^−1^ at the 90th percentile, demonstrating substantial variation in structural benefit per unit mass of installed composite.
2. The CFRP:GFRP material-efficiency ratio was 8.1 (program-clustered 95% CI: 3.8–18.3). As an observational sheet-level benchmark, this ratio defines the cradle-to-gate GHG break-even threshold: GFRP becomes the lower-GHG sheet-level option when the boundary-consistent CFRP:GFRP emission-factor ratio exceeds this threshold. The ratio is an association within the present database rather than a causal material effect; point ratios remained between 7.0 and 8.1 when thickness-convention uncertainty or non-positive contributions were accounted for, and the conservative decision band is 2.8–21.3.
3. Material efficiency generally decreased with increasing installed sheet rigidity, consistent with diminishing structural returns from additional composite material.
4. The response-surface model achieved random ten-fold out-of-fold *R*^2^ = 0.80, decreasing to 0.53 under program-grouped validation and 0.39 under leave-one-program-out validation, indicating substantially weaker transferability to unseen experimental programs. Most of this loss reflected program-level offsets, while within-program ordering and the member-dominated attribution were retained under program-controlled validation.
5. Triangulated attribution showed that member geometry and transverse reinforcement had greater predictive and variance-based importance for the attributed FRP contribution than FRP sheet properties, while Sobol′ analysis attributed approximately 18% of fitted-response variance to interactions.

Overall, the framework complements code-based FRP design as a screening step that follows it. It does not seek the highest material efficiency; among alternatives that already provide the required FRP shear contribution, it identifies those achieving it with the least installed composite, before project-specific environmental and economic assessment.

## Figures and Tables

**Table 1:** Material efficiency, *ME* = *V_f_*_,exp_/*m*, by FRP material and strengthening configuration.

Class	*n*	Median *ME* (kN·m·kg^−1^)	IQR
CFRP	291	303	111–692
AFRP	14	538	440–661
BFRP	7	370	292–500
PET-FRP	10	59	45–79
GFRP	37	38	24–58
Side-bonded	139	175	62–632
U-jacketed	159	304	103–583
Completely wrapped	61	302	53–617
Anchored	90	376	101–708
Unanchored	269	207	67–539

**Table 2:** Break-even material-efficiency ratios with bootstrap 95% confidence intervals.

Comparison	Median *ME* Ratio	95% CI	Interpretation
CFRP:GFRP	8.1	3.8–18.3	GFRP becomes the lower-GHG option if *EF*_CFRP_/*EF*_GFRP_ > 8.1; the same threshold applies to the corresponding price ratio
CFRP:AFRP	0.56	0.38–0.73	AFRP exhibited higher observed median efficiency, but the subset was small (*n* = 14)
CFRP:PET-FRP	5.1	3.3–7.2	PET-FRP becomes the lower-GHG option only if *EF*_CFRP_/*EF*_PET_ > 5.1

**Table 3:** Triangulated importance ranks for the *V_f_*_,exp_ model.

Feature	MI Rank	MI (Norm.)	Perm. Rank	Perm. (Norm.)	±SD	SHAP Rank	SHAP (Norm.)
h	2	0.97	1	1.00	0.060	1	1.00
ρv·fyt	10	0.23	2	0.33	0.040	2	0.51
bw	3	0.97	3	0.18	0.011	3	0.40
Anchorage	14	0.10	4	0.07	0.007	4	0.22
ffu	1	1.00	6	0.04	0.004	5	0.18
f′c	7	0.57	5	0.07	0.008	6	0.12
a/d	8	0.93	8	0.02	0.002	7	0.11
ρs	5	0.66	9	0.02	0.002	8	0.10

## Data Availability

The data supporting the findings of this study are available in the Supplementary Materials and from the corresponding author upon reasonable request. All source experimental programs are cited in the reference list (Refs. [32,33,34,35,36,37,38,39,40,41,42,43,44,45,46,47,48,49,50,51,52,53,54,55,56,57,58,59,60,61,62,63,64,65,66,67,68,69,70,71,72,73,74,75,76]) and listed in Table A1.

## Supplementary Materials

The following supporting information can be downloaded at: https://www.mdpi.com/article/10.3390/polym18202503/s1, References [10,12,15,17,19,32,33,34,35,36,37,38,39,40,41,42,43,44,45,46,47,48,49,50,51,52,53,54,55,56,57,58,59,60,61,62,63,64,65,66,67,68,69,70,71,72,73,74,75,76] are cited in the Supplementary Materials.

## Appendix A. Source Programs and Representative Strengthening Schemes

Table A1 lists the 45 published sources considered; 43 experimental programs contributed the 359 retained specimen records. Table A2 presents one representative strengthening scheme for each principal category of the database; for each category, the specimen with *a*/*d* ≥ 2 whose material efficiency was closest to the category median is shown.

**Table A1:** Published sources constituting the database.

Ref.	Program	Year	*n* (Excl.)	FRP	Scheme	Section	Anchored	Pre-Cracked
[32]	Abdel-Jaber et al.	2003	7	CFRP	SB/UJ	R	0	0
[33]	Adhikary and Mutsuyoshi	2004	3	CFRP	SB/UJ	R	0	0
[34]	Allam and Ebeido	2003	11	CFRP	SB/UJ	R	0	7
[35]	Beber and Campos Filho	2005	22	CFRP	SB/UJ/CW	R	0	0
[36]	Bukhari et al.	2010	6	CFRP	SB/CW	R	0	0
[37]	Täljsten	2003	5	CFRP	SB	R	0	1
[38]	Carolin and Täljsten	2005	13	CFRP	SB/CW	R	3	3
[39]	Chaallal et al.	1998	4	CFRP	SB	R	0	0
[40]	Grande et al.	2009	10	CFRP	SB/UJ/CW	R	3	0
[41]	Pellegrino and Modena	2002	8 (1)	CFRP	SB	R	0	0
[42]	Cao et al.	2005	12	CFRP/GFRP	CW	R	0	12
[43]	Ianniruberto and Imbimbo	2004	3 (1)	GFRP	CW	R	0	0
[44]	Zhang and Hsu	2005	6	CFRP	SB	R	0	0
[45]	Al-Shamayleh et al.	2022	6	CFRP	SB/UJ	R	0	0
[46]	Ibrahim et al.	2021	6 (2)	CFRP	UJ	T	0	0
[47]	Sarıbıyık et al.	2021	7	BFRP	UJ/CW	R	3	0
[48]	Oller et al.	2019	14 (2)	CFRP	UJ	T	8	0
[49]	Mofidi and Chaallal	2014	10	CFRP	UJ	T	0	0
[50]	Foster et al.	2017	6 (3)	CFRP	UJ	T	3	0
[51]	Beramly et al.	2022	4	CFRP	SB/UJ	T	0	2
[52]	Jiang et al.	2025	5	CFRP	SB	T	4	2
[53]	Dias and Barros	2010	12	CFRP	SB/UJ	T	0	0
[54]	Barros et al.	2007	8	CFRP	SB	R	0	0
[55]	Ebead and Saeed	2013	19	CFRP	SB	R	13	0
[56]	Hadhood et al.	2019	7	CFRP	SB	R	6	0
[57]	Zhou et al.	2019	4	CFRP	UJ	R	3	0
[58]	Karzad et al.	2019	12	CFRP	UJ	R	0	6
[59]	Li and Leung	2016	6	CFRP	CW	R	0	0
[60]	Mosallam and Banerjee	2007	6	CFRP/GFRP	SB/UJ	R	0	3
[61]	Mei et al.	2024	10	PET-FRP	CW	R	0	0
[62]	Umezu et al.	1997	15	CFRP/AFRP	UJ/CW	R	0	0
[63]	Asrianur et al.	2026	11 (1)	CFRP	SB/UJ	T	6	0
[64]	Deniaud and Cheng	2003	4	CFRP/GFRP	UJ	T	0	0
[65]	Chaallal et al.	2002	9	CFRP/AFRP	UJ/CW	T	0	0
[67]	Panda et al.	2011	9	GFRP	SB	T	0	0
[68]	El-Saikaly et al.	2015	9	CFRP	UJ	T	3	0
[69]	Kim et al.	2015	0 (7)	–	–	–	–	–
[70]	Panda et al.	2013	9	GFRP	SB/UJ	T	3	0
[71]	Panigrahi et al.	2016	3	GFRP	UJ	T	0	0
[72]	Randl and Harsányi	2018	6	CFRP	UJ	T	6	6
[73]	Belarbi et al.	2012	6	CFRP	UJ	T	4	2
[74]	Chen et al.	2016	9	CFRP	UJ	T	7	0
[75]	Kim et al.	2014	11	CFRP	UJ	T	9	2
[76]	Bourget et al.	2017	6	CFRP	UJ	T	6	0

*n* = specimens retained in the analysis; excluded specimens (non-positive or undetermined *V_f_*_,exp_) in parentheses. SB = side-bonded; UJ = U-jacketed; CW = completely wrapped; R = rectangular; T = T-section. Ref. [69] contributed only specimens with undetermined *V_f_*_,exp_.

**Table A2:** Representative strengthening schemes by category.

Category	*n*	Median *ME* (IQR)	Specimen [Ref.]	Member	FRP Layout	*V_f_*_,exp_/*m*/*ME*	Failure Mode
CFRP · side-bonded · rect. · unanchored	87	219 (121–725)	RS4Sb [40]	R, 250 × 450 mm; *a*/*d* = 3.4; *ρ_v_f_yt_* = 0.48 MPa	0.191 mm × 1; *w_f_*/*s_f_* = 1.00; side-bonded	55.0/0.251/219	FRP edge debonding
CFRP · U-jacket · T-sec. · unanchored	49	113 (69–256)	JS4A [65]	T, 152 × 381 mm; *a*/*d* = 2.6; no stirrups	0.33 mm × 1; *w_f_*/*s_f_* = 1.00; U-jacket	45.5/0.402/113	Debonding
CFRP · U-jacket · T-sec. · anchored	49	506 (309–754)	S0-LS-RF [76]	T, 152 × 406 mm; *a*/*d* = 3.0; no stirrups	2 mm × 1; *w_f_*/*s_f_* = 0.11; U-jacket; anchored	120.0/0.237/506	Shear with anchorage failure
CFRP · complete wrap · rect.	21	764 (397–1041)	V12_A [35]	R, 150 × 300 mm; *a*/*d* = 3.0; no stirrups	0.111 mm × 1; *w_f_*/*s_f_* = 0.50; complete wrap	59.4/0.080/744	CFRP rupture
CFRP · pre-cracked	37	491 (230–816)	A3 [42]	R, 150 × 250 mm; *a*/*d* = 2.7; *ρ_v_f_yt_* = 0.57 MPa; pre-cracked	0.167 mm × 1; *w_f_*/*s_f_* = 0.20; complete wrap	21.0/0.043/491	FRP rupture near lower beam corner
GFRP · side-bonded · T-sec.	12	42 (33–64)	S200-1L-CT-S-90 [67]	T, 100 × 260 mm; *a*/*d* = 3.3; *ρ_v_f_yt_* = 0.71 MPa	0.36 mm × 1; *w_f_*/*s_f_* = 1.00; side-bonded	10.0/0.238/42	GFRP debonding
GFRP · complete wrap · rect.	11	28 (24–40)	L2 [42]	R, 150 × 250 mm; *a*/*d* = 2.9; no stirrups; pre-cracked	1.2 mm × 1; *w_f_*/*s_f_* = 0.50; complete wrap	26.0/0.960/27	FRP rupture near mid-height of beam
AFRP (all)	14	538 (440–661)	AB3 [62]	R, 300 × 300 mm; *a*/*d* = 3.0; no stirrups	0.088 mm × 1; *w_f_*/*s_f_* = 1.00; complete wrap	81.6/0.148/552	FRP rupture
BFRP (all)	7	370 (292–500)	USHA90 [47]	R, 150 × 250 mm; *a*/*d* = 2.5; *ρ_v_f_yt_* = 1.22 MPa	0.14 mm × 1; *w_f_*/*s_f_* = 0.60; U-jacket; anchored	30.7/0.083/370	Shear with FRP rupture
PET-FRP (all)	10	59 (45–79)	B2W70 [61]	R, 150 × 300 mm; *a*/*d* = 2.2; *ρ_v_f_yt_* = 0.80 MPa	0.841 mm × 1; *w_f_*/*s_f_* = 0.47; complete wrap	30.6/0.477/64	Ductile shear failure; no FRP rupture or debonding

*ME* in kN·m·kg^−1^; *V_f_*_,exp_ in kN; *m* in kg·m^−1^; FRP layout gives ply thickness × number of plies, strip-continuity ratio, and wrapping scheme. rect. = rectangular section; T-sec. = T-section.
