# Supplementary material for: Material-Efficient Selection of Externally Bonded Fiber-Reinforced Polymer Composites for RC Shear Strengthening Using a Literature-Based Database and Break-Even Criteria
Source: Polymers (Basel). 2026 Oct 13;18(20):2503. doi: 10.3390/polym18202503 (PMC13708974; doi:10.3390/polym18202503)
Supplement: Supplementary file 1 [file polymers-18-02503-s001.zip › polymers-4593134-supplementary.pdf]

Supplementary Material

Material-Efficient Selection of Externally Bonded Fiber-Reinforced Polymer Composites for RC Shear Strengthening Using a Literature-Based Database and Break-Even Criteria

This Supplementary Material provides the database source bibliography, FRP mass calculations, response-surface model hyperparameters, complete sensitivity and ablation results, and uncertainty analyses supporting the main article. Programme-controlled validation diagnostics, target-definition robustness, stratified material-efficiency comparisons, subgroup predictive performance, signed-efficiency exclusion analyses, thickness-convention and density uncertainty, and extensions of the cradle-to-gate environmental boundary are also reported.

Contents

S1. Database source bibliography 2
S2. FRP mass and efficiency calculations 6
S3. Response-surface model hyperparameters 7
S4. Sobol′ variance decomposition 8
S5. Group-ablation analysis 9
S6. Uncertainty-analysis details 10
S7. Programme-grouped cross-validation 12
S8. Robustness to target normalization 14
S9. Stratified material-efficiency comparison 15
S10. Subgroup predictive performance 16
S11. Excluded specimens and exclusion sensitivity 17
S12. Environmental boundary matching 19

S1. Database source bibliography

The principal analysis contains 359 specimens from 43 experimental programmes. Table S1-1 lists all 45 published sources considered. Source [38] (main-text Ref. [69]) contributed only specimens with undetermined FRP-attributed shear contribution and therefore no retained record. Source numbers in Table S1-1 correspond to main-text Refs. [32]–[76].

A narrower single-programme database would likely produce higher apparent predictive accuracy because specimens would share concrete batches, fabrication procedures, test setups, and measurement practices. However, such a database would provide only a limited basis for cross-material and cross-configuration comparison. The present study therefore accepts a deliberate trade-off between predictive precision and design-space coverage: the heterogeneous database enables comparison of fibre systems and strengthening configurations on a common basis, while programme-grouped validation is subsequently used to quantify transferability to unseen experimental programmes.

Table S1-1. Experimental programmes constituting the database

1. Abdel-Jaber, M.S.; Walker, P.R.; Hutchinson, A.R. Shear strengthening of reinforced concrete beams using different configurations of externally bonded carbon fibre reinforced plates. *Mater. Struct.* 2003, 36, 291–301. https://doi.org/10.1007/BF02480868.

2. Adhikary, B.B.; Mutsuyoshi, H. Behavior of concrete beams strengthened in shear with carbon-fiber sheets. *J. Compos. Constr.* 2004, 8, 258–264. https://doi.org/10.1061/(ASCE)1090-0268(2004)8:3(258).

3. Allam, S.M.; Ebeido, T.I. Retrofitting of RC beams predamaged in shear using CFRP sheets. *Alex. Eng. J.* 2003, 42, 87–101.

4. Beber, A.J.; Campos Filho, A. CFRP composites on the shear strengthening of reinforced concrete beams. *Rev. IBRACON Estrut.* 2005, 1, 127–143.

5. Bukhari, I.A.; Vollum, R.L.; Ahmad, S.; Sagaseta, J. Shear strengthening of reinforced concrete beams with CFRP. *Mag. Concr. Res.* 2010, 62, 65–77. https://doi.org/10.1680/macr.2008.62.1.65.

6. Täljsten, B. Strengthening concrete beams for shear with CFRP sheets. *Constr. Build. Mater.* 2003, 17, 15–26. https://doi.org/10.1016/S0950-0618(02)00088-0.

7. Carolin, A.; Täljsten, B. Experimental study of strengthening for increased shear bearing capacity. *J. Compos. Constr.* 2005, 9, 488–496. https://doi.org/10.1061/(ASCE)1090-0268(2005)9:6(488).

8. Chaallal, O.; Nollet, M.-J.; Perraton, D. Shear strengthening of RC beams by externally bonded side CFRP strips. *J. Compos. Constr.* 1998, 2, 111–113. https://doi.org/10.1061/(ASCE)1090-0268(1998)2:2(111).

9. Grande, E.; Imbimbo, M.; Rasulo, A. Effect of transverse steel on the response of RC beams strengthened in shear by FRP: Experimental study. *J. Compos. Constr.* 2009, 13, 405–414. https://doi.org/10.1061/(ASCE)1090-0268(2009)13:5(405).

10. Pellegrino, C.; Modena, C. Fiber reinforced polymer shear strengthening of reinforced concrete beams with transverse steel reinforcement. *J. Compos. Constr.* 2002, 6, 104–111. https://doi.org/10.1061/(ASCE)1090-0268(2002)6:2(104).

11. Cao, S.Y.; Chen, J.F.; Teng, J.G.; Hao, Z.; Chen, J. Debonding in RC beams shear strengthened with complete FRP wraps. *J. Compos. Constr.* 2005, 9, 417–428. https://doi.org/10.1061/(ASCE)1090-0268(2005)9:5(417).

12. Iannirubert‍o, U.; Imbimbo, M. Role of fiber reinforced plastic sheets in shear response of reinforced concrete beams: Experimental and analytical results. *J. Compos. Constr.* 2004, 8, 415–424. https://doi.org/10.1061/(ASCE)1090-0268(2004)8:5(415).

13. Zhang, Z.; Hsu, C.-T.T. Shear strengthening of reinforced concrete beams using carbon-fiber-reinforced polymer laminates. *J. Compos. Constr.* 2005, 9, 158–169. https://doi.org/10.1061/(ASCE)1090-0268(2005)9:2(158).

14. Al-Shamayleh, R.; Al-Saoud, H.; Abdel-Jaber, M.; Alqam, M. Shear and flexural strengthening of reinforced concrete beams with variable compressive strength values using externally bonded carbon fiber plates. *Results Eng.* 2022, 14, 100427. https://doi.org/10.1016/j.rineng.2022.100427.

15. Ibrahim, B.; Leblouba, M.; Altoubat, S.; Barakat, S. Shear strength of externally U-bonded carbon fiber-reinforced polymer high-strength reinforced concrete. *Materials* 2021, 14, 3659. https://doi.org/10.3390/ma14133659.

16. Sarıbıyık, A.; Abodan, B.; Balci, M.T. Experimental study on shear strengthening of RC beams with basalt FRP strips using different wrapping methods. *Eng. Sci. Technol. Int. J.* 2021, 24, 192–204. https://doi.org/10.1016/j.jestch.2020.06.003.

17. Oller, E.; Pujol, M.; Marí, A.R. Contribution of externally bonded FRP shear reinforcement to the shear strength of RC beams. *Compos. Part B Eng.* 2019, 164, 235–248. https://doi.org/10.1016/j.compositesb.2018.11.065.

18. Mofidi, A.; Chaallal, O. Tests and design provisions for reinforced-concrete beams strengthened in shear using FRP sheets and strips. *Int. J. Concr. Struct. Mater.* 2014, 8, 117–128. https://doi.org/10.1007/s40069-013-0060-1.

19. Foster, R.M.; Brindley, M.; Lees, J.M.; Ibell, T.J.; Morley, C.T.; Darby, A.P.; Evernden, M.C. Experimental investigation of reinforced concrete T-beams strengthened in shear with externally bonded CFRP sheets. *J. Compos. Constr.* 2017, 21, 04016086. https://doi.org/10.1061/(ASCE)CC.1943-5614.0000743.

20. Beramly, M.; Abdel-Jaber, M.; Katkhuda, H.; Shatarat, N.; Al-Diseet, M. Shear strengthening and rehabilitating of reinforced concrete T-beams using externally carbon fiber reinforced polymer sheets. *J. Appl. Eng. Sci.* 2022, 20, 498–510. https://doi.org/10.5937/jaes0-34390.

21. Jiang, C.; Xiong, W.; Fu, Y.; Ye, J. Experimental investigation on shear performance of RC T-beams strengthened with hybrid bonded CFRP strips. *Eng. Fail. Anal.* 2025, 167, 109080. https://doi.org/10.1016/j.engfailanal.2024.109080.

22. Dias, S.J.E.; Barros, J.A.O. Performance of reinforced concrete T beams strengthened in shear with NSM CFRP laminates. *Eng. Struct.* 2010, 32, 373–384. https://doi.org/10.1016/j.engstruct.2009.10.001.

23. Barros, J.A.O.; Dias, S.J.E.; Lima, J.L.T. Efficacy of CFRP-based techniques for the flexural and shear strengthening of concrete beams. *Cem. Concr. Compos.* 2007, 29, 203–217. https://doi.org/10.1016/j.cemconcomp.2006.09.001.

24. Ebead, U.; Saeed, H. Hybrid shear strengthening system for reinforced concrete beams: An experimental study. *Eng. Struct.* 2013, 49, 421–433. https://doi.org/10.1016/j.engstruct.2012.11.039.

25. Hadhood, A.; Agamy, M.H.; Abdelsalam, M.M.; Mohamed, H.M.; Elsayed, T.A. Shear strengthening of hybrid externally-bonded mechanically-fastened concrete beams using short CFRP strips: Experiments and theoretical evaluation. *Eng. Struct.* 2019, 201, 109795. https://doi.org/10.1016/j.engstruct.2019.109795.

26. Zhou, Y.; Guo, M.; Sui, L.; Xing, F.; Hu, B.; Huang, Z.; Yun, Y. Shear strength components of adjustable hybrid bonded CFRP shear-strengthened RC beams. *Compos. Part B Eng.* 2019, 163, 36–51. https://doi.org/10.1016/j.compositesb.2018.11.020.

27. Karzad, A.S.; Leblouba, M.; Al Toubat, S.; Maalej, M. Repair and strengthening of shear-deficient reinforced concrete beams using carbon fiber reinforced polymer. *Compos. Struct.* 2019, 223, 110963. https://doi.org/10.1016/j.compstruct.2019.110963.

28. Li, W.; Leung, C.K.Y. Shear span–depth ratio effect on behavior of RC beam shear strengthened with full-wrapping FRP strip. *J. Compos. Constr.* 2016, 20, 04015067. https://doi.org/10.1061/(ASCE)CC.1943-5614.0000627.

29. Mosallam, A.S.; Banerjee, S. Shear enhancement of reinforced concrete beams strengthened with FRP composite laminates. *Compos. Part B Eng.* 2007, 38, 781–793. https://doi.org/10.1016/j.compositesb.2006.10.002.

30. Mei, S.-J.; Bai, Y.-L.; Dai, J.-G. Shear strengthening of reinforced concrete beams completely wrapped by large rupture strain (LRS) FRP. *Eng. Struct.* 2024, 314, 118361. https://doi.org/10.1016/j.engstruct.2024.118361.

31. Umezu, K.; Fujita, M.; Nakai, H.; Tamaki, K. Shear behavior of RC beams with aramid fiber sheet. In Proceedings of the Third International Symposium on Non-Metallic (FRP) Reinforcement for Concrete Structures (FRPRCS-3), Sapporo, Japan, 14–16 October 1997; Japan Concrete Institute: Tokyo, Japan, 1997; Volume 1, pp. 491–498.

32. Asrianur, A.; Thamrin, R.; Haris, S.; Kurniawan, R. An evaluation of the shear capacity of strengthened reinforced concrete T-beams with inverted U-wrap or completely wrapped using CFRP anchors. *Next Mater.* 2026, 12, 102262. https://doi.org/10.1016/j.nxmate.2026.102262.

33. Deniaud, C.; Cheng, J.J.R. Reinforced concrete T-beams strengthened in shear with fiber reinforced polymer sheets. *J. Compos. Constr.* 2003, 7, 302–310. https://doi.org/10.1061/(ASCE)1090-0268(2003)7:4(302).

34. Chaallal, O.; Shahawy, M.; Hassan, M. Performance of reinforced concrete T-girders strengthened in shear with carbon fiber-reinforced polymer fabric. *ACI Struct. J.* 2002, 99, 335–343. https://doi.org/10.14359/11917.

35. Gamino, A.L.; Sousa, J.L.A.O.; Manzoli, O.L.; Bittencourt, T.N. Estruturas de concreto reforçadas com PRFC Parte II: análise dos modelos de cisalhamento. *Rev. IBRACON Estrut. Mater.* 2010, 3, 24–49. https://doi.org/10.1590/S1983-41952010000100003.

36. Panda, K.C.; Bhattacharyya, S.K.; Barai, S.V. Shear strengthening of RC T-beams with externally side bonded GFRP sheet. *J. Reinf. Plast. Compos.* 2011, 30, 1139–1154. https://doi.org/10.1177/0731684411417202.

37. El-Saikaly, G.; Godat, A.; Chaallal, O. New anchorage technique for FRP shear-strengthened RC T-beams using CFRP rope. *J. Compos. Constr.* 2015, 19, 04014064. https://doi.org/10.1061/(ASCE)CC.1943-5614.0000530.

38. Kim, Y.; Ghannoum, W.M.; Jirsa, J.O. Shear behavior of full-scale reinforced concrete T-beams strengthened with CFRP strips and anchors. *Constr. Build. Mater.* 2015, 94, 1–9. https://doi.org/10.1016/j.conbuildmat.2015.06.005.

39. Panda, K.C.; Bhattacharyya, S.K.; Barai, S.V. Effect of transverse steel on the performance of RC T-beams strengthened in shear zone with GFRP sheet. *Constr. Build. Mater.* 2013, 41, 79–90. https://doi.org/10.1016/j.conbuildmat.2012.11.098.

40. Panigrahi, S.K.; Deb, A.; Bhattacharyya, S.K. Modes of failure in shear deficient RC T-beams strengthened with FRP. *J. Compos. Constr.* 2016, 20, 04015029. https://doi.org/10.1061/(ASCE)CC.1943-5614.0000586.

41. Randl, N.; Harsányi, P. Developing optimized strengthening systems for shear-deficient concrete members. *Struct. Concr.* 2018, 19, 116–128. https://doi.org/10.1002/suco.201600187.

42. Belarbi, A.; Bae, S.-W.; Brancaccio, A. Behavior of full-scale RC T-beams strengthened in shear with externally bonded FRP sheets. *Constr. Build. Mater.* 2012, 32, 27–40. https://doi.org/10.1016/j.conbuildmat.2010.11.102.

43. Chen, G.M.; Zhang, Z.; Li, Y.L.; Li, X.Q.; Zhou, C.Y. T-section RC beams shear-strengthened with anchored CFRP U-strips. *Compos. Struct.* 2016, 144, 57–79. https://doi.org/10.1016/j.compstruct.2016.02.033.

44. Kim, Y.; Quinn, K.; Ghannoum, W.M.; Jirsa, J.O. Strengthening of reinforced concrete T-beams using anchored CFRP materials. *ACI Struct. J.* 2014, 111, 1027–1036. https://doi.org/10.14359/51687025.

45. Bourget, S.; El-Saikaly, G.; Chaallal, O. Behavior of reinforced concrete T-beams strengthened in shear using closed carbon fiber-reinforced polymer stirrups made of laminates and ropes. *ACI Struct. J.* 2017, 114, 1087–1098. https://doi.org/10.14359/51700786.

S2. FRP mass and efficiency calculations

The installed FRP mass was calculated for each specimen using the reported sheet geometry, number of plies, wrapping configuration, strip continuity, and adopted cured-composite density.

Table S2-1. Quantities calculated for each specimen

Units: N and mm; mass expressed per meter of strengthened zone.

Quantity	Expression or adopted value
Bonded perimeter, p	$2d_{fv}$ for SB; $2d_{fv}+b_w$ for UJ; $2(h+b_w)$ for CW
Sheet volume per meter	$t_f n(w_f/s_f)p$
ρ_{FRP}	1.60 for CFRP; 2.00 for GFRP; 1.40 for AFRP; 2.10 for BFRP; 1.35 for PET-FRP, in $g\cdot cm^{-3}$
Installed mass per meter, m	$t_f n(w_f/s_f)p\rho_{FRP}\times 10^{-3}$, in $kg\cdot m^{-1}$
Material efficiency	$ME=V_{f,exp}/m$, in $kN\cdot m\cdot kg^{-1}$
Break-even rule for systems A and B	Within the adopted cradle-to-gate boundary, system B is the lower-GHG option when $EF_A/EF_B>ME_A/ME_B$

The installed-mass term excludes adhesive, primer, putty, anchors, and installation consumables. The adopted environmental boundary is cradle-to-gate production of the cured FRP-composite sheet. The adopted densities represent typical cured-composite values because specimen-specific composite densities and fibre-volume fractions were not reported consistently across the source programmes. Reported sheet thickness also follows inconsistent conventions across studies; the resulting thickness-convention and density uncertainty is propagated explicitly in Section S6.

S3. Response-surface model hyperparameters

Hyperparameters for the response-surface model predicting $V_{f,\mathrm{exp}}$were selected using a randomized search over 40 parameter combinations with internal five-fold cross-validation. The optimization objective was the coefficient of determination R^2. All performance values reported in the main article and Supplementary Material were subsequently calculated using a separate ten-fold out-of-fold protocol with shuffled folds and a fixed random seed of 42.

Table S3-1. Hyperparameter search space and selected values for the response-surface model predicting $V_{f,exp}$

Hyperparameter	Search space	Selected value
n_estimators	200, 400, 600, 800	200
max_depth	3–6	3
learning_rate	0.02–0.10	0.02
subsample	0.70, 0.85, 1.00	0.70
colsample_bytree	0.70, 0.85, 1.00	0.85
min_child_weight	1, 3, 5	5
reg_lambda	0.5, 1.0, 2.0	2.0

Stirrup yield strength, fyt, was unreported for 72 specimens in the parent database. All 72 were members without transverse reinforcement (Asv = ρ_v = 0), so fyt entered the response-surface model only through the product $\rho_v \cdot f_{yt}$ and was multiplied by zero in every case; these observations were assigned the median value of 414 MPa observed for stirrup-bearing specimens. Re-running the model with imputed values of 200, 414, and 800 MPa returned an identical out-of-fold R^2 of 0.8015, confirming that the imputation did not influence any reported result. Out-of-fold predictive performance was nonetheless lower for this subset (R^2 = 0.708, n = 72 in the analysis set) than for stirrup-bearing specimens (R^2 = 0.818, n = 287), reflecting the greater variability of members without transverse reinforcement rather than any effect of the imputation.

S4. Sobol′ variance decomposition

Sobol′ variance decomposition was applied to the fitted V_f response surface using Saltelli sampling with 4096 base samples. Second-order estimation was enabled. Binary variables were sampled discretely, and the wrapping-scheme indicators were constrained to remain mutually exclusive. Continuous variables were sampled over the 1st–99th percentile of their observed ranges.

Table S4-1. Sobol′ first-order and total-order indices of the $V_{f,exp}$ response surface

Feature	First-order index, S_i	Total-order index, S_{Ti}
h	0.311	0.485
$\rho_v f_{yt}$	0.317	0.431
b_w	0.076	0.090
f_c'	0.039	0.089
Anchorage	0.045	0.048
CW	0.010	0.016
f_{fu}	0.007	0.011
w_f/s_f	0.007	0.011
ρ_s	0.005	0.006

The summed first-order indices were 0.821, indicating that approximately 18% of the fitted-response variance was not captured by first-order effects and was therefore attributable primarily to interaction effects under the adopted independent-input sampling scheme. Because Sobol′ analysis assumes independent inputs, these indices characterize the fitted response surface over the sampled synthetic design domain rather than the correlated joint distribution of the experimental database. Table S5-1 provides the complementary predictive interpretation based on group ablation. The complementary roles of the variance-based and predictive perspectives are discussed in Section 4.3 of the main article.

S5. Group-ablation analysis

Group ablation was performed by removing each mechanically defined feature block, retraining the response-surface model, and repeating the complete ten-fold out-of-fold evaluation. The baseline out-of-fold coefficient of determination was $R^2 = 0.802$.

Table S5-1. Out-of-fold R^2 after removal of each feature group

**Removed feature group**	R^2 **without group**	ΔR^2
FRP-properties group	0.799	0.002
Transverse-reinforcement group	0.761	0.040
Strengthening-configuration group	0.779	0.022
Member-geometry group	0.715	0.087
Longitudinal-reinforcement group	0.800	0.002

The largest predictive loss occurred after removing the geometry block, followed by the transverse-reinforcement block and the strengthening-configuration block. Removing the complete FRP-properties group caused only a small reduction in out-of-fold accuracy, indicating that the group provided little unique predictive information beyond that contained in the remaining predictor groups.

S6. Uncertainty-analysis details

Break-even ratios were calculated as ratios of median material efficiencies. Specimen-level uncertainty was first quantified by 5000 nonparametric bootstrap resamples (seed 1). Because specimens within an experimental programme share materials, fabrication, instrumentation, and sometimes a common companion control, the principal CFRP:GFRP interval was obtained using a two-stage programme-clustered bootstrap: programmes were resampled with replacement and specimens were then resampled within each selected programme. The specimen-level 95% CI was 5.6–11.9, whereas the programme-clustered CI widened to 3.8–18.3 and was adopted as the primary sampling interval. Measurement- and convention-related uncertainty in installed mass was then propagated by Monte Carlo simulation nested within the programme-clustered bootstrap (5000 replicates, seed 1). A programme-level factor represented thickness convention and a material-level factor represented cured-composite density (CFRP 1.45–1.75 $g \cdot cm^{-3}$; GFRP 1.70–2.20 $g \cdot cm^{-3}$). In the convention-informed model, reported thicknesses ≤0.45 mm (185 CFRP and 23 GFRP specimens) were treated as dry-fiber-equivalent values and converted to cured-laminate mass using a fiber-volume fraction sampled uniformly from 0.30–0.60; thicker products received a factor of 0.85–1.15. A convention-agnostic stress test assigned every programme a log-uniform thickness factor of 0.5–2.0. Results are summarized in Table S6-1. The central ratio remained stable, whereas the combined stress-test interval widened to 2.8–21.3; these bounds are therefore used as the conservative screening band in the main article.

Table S6-1. CFRP:GFRP break-even ratio under sampling, thickness-convention, and cured-composite-density uncertainty.

**Uncertainty treatment**	**CFRP:GFRP ratio**	**95% interval / range**
Specimen-level bootstrap	8.1	5.6–11.9
Programme-clustered bootstrap	8.1	3.8–18.3
Convention-informed + density uncertainty only	7.3	5.7–9.3
Convention-informed + programme sampling	—	3.7–16.5
Convention-agnostic stress test + density uncertainty only	7.8	4.7–12.6
Convention-agnostic stress test + programme sampling	—	2.8–21.3
Deterministic harmonization of thin-sheet thickness	7.0–7.9	point-ratio range

The retained material-subset sizes were:

- CFRP: $n = 291$
- GFRP: $n = 37$
- AFRP: $n = 14$
- BFRP: $n = 7$
- PET-FRP: $n = 10$

Ratios involving AFRP, BFRP, and PET-FRP should be interpreted cautiously because of the limited sample sizes. Because the material-efficiency distributions were strongly right-skewed, medians and interquartile ranges were used throughout, and no parametric distribution was assumed.

S7. Programme-grouped cross-validation

The principal predictive results reported in the main article were obtained using shuffled ten-fold cross-validation. Under this protocol, specimens from the same experimental programme may appear in both the training and test folds. To assess transferability to previously unseen experimental programmes, the model was re-evaluated using (i) ten-fold programme-grouped cross-validation, in which all specimens from a given programme were assigned to the same fold, and (ii) leave-one-programme-out validation, in which each of the 43 represented programmes was withheld in turn.

Hyperparameters for the reported models were initially selected once on the assembled database. As a verification step, the entire workflow was repeated using nested cross-validation, with the randomized hyperparameter search performed independently within each outer training fold. For the programme-controlled validation protocols, the inner hyperparameter search also respected programme grouping. Nested evaluation produced $R^2 = 0.797$ for random ten-fold validation and $R^2 = 0.507$ for programme-grouped validation, closely consistent with the corresponding values obtained using the fixed hyperparameters and indicating that hyperparameter selection outside the outer folds did not materially inflate the reported predictive performance.

Predictive performance decreased substantially under both programme-controlled protocols (Table S7-1), indicating reduced generalization to previously unseen experimental programmes. Under programme-grouped cross-validation, fold-specific R^2 values ranged from −1.40 to 0.74 (median 0.31). Under leave-one-programme-out validation, R^2 was negative for 33 of the 43 held-out programmes, although individual values are sensitive to small sample size and limited within-programme response variance. The transfer loss was therefore decomposed further (Table S7-2). Differences between programme means account for 74% of the variance of $V_{f,exp}$. Under leave-one-programme-out validation, 64% of mean squared error arose from the programme-level mean residual, compared with 12% under random cross-validation; programme means were reproduced with R^2 = 0.52. Within-programme ordering was more stable: the median Spearman correlation between observed and predicted contributions was 0.45 and was positive in 32 of the 40 programmes containing at least four specimens. Thus, transfer loss is dominated by programme-level offsets rather than complete loss of within-programme ordering.

Table S7-1. Out-of-fold accuracy under random, programme-grouped, and leave-one-programme-out validation

**Validation protocol**	R^2	**RMSE (kN)**
Random ten-fold, shuffled, seed 42	0.802	30.4
Programme-grouped ten-fold, GroupKFold	0.526	47.0
Leave-one-programme-out, 43 programmes	0.394	53.1

Table S7-2. Diagnostics of programme-level transferability.

**Diagnostic**	**Result**
Between-programme share of $V_{f,exp}$ variance	74%
Programme-level offset share of MSE: random ten-fold	12%
Programme-level offset share of MSE: leave-one-programme-out	64%
R^2 for programme means	0.52
Median within-programme Spearman ρ	0.45
Positive within-programme ρ	32 of 40 programmes with $n \geq 4$

Attribution robustness was also examined under programme-controlled validation. Removing member geometry reduced R^2 by 0.43 under programme-grouped validation and by 0.34 under leave-one-programme-out validation; removing transverse reinforcement reduced R^2 by 0.13 and 0.31, respectively. By contrast, removing the FRP-properties group slightly improved transfer ($\Delta R^2 = -0.04$ and −0.09). Across the 43 leave-one-programme-out models, varying beam depth over its 10th–90th-percentile range at the Fig. 6 reference configuration changed predicted contribution by a median of 51 kN, compared with 0.3 kN for sheet rigidity and 2.3 kN for strip continuity; the beam-depth effect exceeded both sheet-property effects in all 43 models. These results support the member-dominated attribution while confirming that absolute map levels remain conditional on programme context.

Table S7-3. Programme-controlled attribution and partial-dependence robustness.

**Removed group / perturbation**	**Programme-grouped**	**Leave-one-programme-out**
Member geometry, ΔR^2	0.43	0.34
Transverse reinforcement, ΔR^2	0.13	0.31
FRP properties, ΔR^2	−0.04	−0.09
Median partial-dependence span: beam depth	—	51 kN
Median partial-dependence span: sheet rigidity	—	0.3 kN
Median partial-dependence span: strip continuity	—	2.3 kN

S8. Robustness to target normalization

The primary modelling target was the absolute experimentally attributed FRP contribution, $V_{f,\mathrm{exp}}$, in kilonewtons. Because larger members generally develop larger absolute contributions, geometric variables may dominate partly through response scaling.

The response-surface model was therefore repeated using:

$$\frac{V_{f,\mathrm{exp}}}{b_w d}$$

and

$$\log\left(V_{f,\mathrm{exp}}\right)$$

as alternative targets. Permutation importance was additionally evaluated for the size-normalized response to assess whether the prominence of geometric variables was driven by first-order member-size scaling.

The normalized response removes first-order member-size scaling while retaining f_c' the geometric predictors as explicit model inputs rather than embedding them within the response. Under this normalization, the leading position of beam depth h weakened and the stirrup index became the dominant permutation variable, followed by longitudinal reinforcement, anchorage, a/d, beam depth, and d_{fv}/d. The result indicates that part of the prominence of h in the absolute-response model was associated with member-size scaling, while member, reinforcement, and configuration variables remained prominent relative to the principal FRP-sheet properties.

Table S8-1. Out-of-fold R^2 and leading permutation variables under alternative targets

Target	Out-of-fold R^2	Permutation-importance order, top six
Absolute $V_{f,\mathrm{exp}}$, kN	0.802	h, $\rho_v f_{yt}$, b_w, f_c', Anchorage, f_{fu}
$V_{f,\mathrm{exp}}/(b_w d)$	0.520	$\rho_v f_{yt}$, ρ_s, Anchorage, a/d, h, d_{fv}/d
$\log\left(V_{f,\mathrm{exp}}\right)$	0.655	Not reported

S9. Stratified material-efficiency comparison

The CFRP and GFRP subsets differed in wrapping scheme, section type, anchorage, and source programme. The overall CFRP:GFRP material-efficiency ratio should therefore be interpreted as an observational database contrast rather than as a controlled causal material effect.

To examine whether the overall contrast was driven by individual major configuration variables, the ratio of median material efficiency was recalculated within matched strata. The ratio remained between 7.5 and 10.9 across the structural subsets. Product form was examined separately using the reported-thickness convention adopted for the uncertainty analysis: thin sheets and fabrics (tf ≤ 0.45 mm) and cured laminates and plates (tf > 0.45 mm). CFRP retained the higher median efficiency in both product-form strata, with ratios of 9.9 and 4.8, respectively. This persistence indicates that the overall contrast is not explained solely by one examined configuration variable or by product form. However, the observed CFRP–GFRP efficiency contrast should not be interpreted as a causal material effect, because differences in other programme- and specimen-level characteristics may remain even within the matched structural subsets.

Table S9-1. CFRP:GFRP median material-efficiency ratios within selected strata

**Stratum**	n_C	n_G	**Median CFRP** ME	**Median GFRP** ME	**Ratio**
Overall, unadjusted	291	37	303	38	8.07
U-jacketed only	139	14	336	36	9.33
Rectangular sections only	168	13	296	27	10.92
Unanchored systems only	207	34	269	36	7.54
Thin sheets/fabrics (tf ≤ 0.45 mm)	185	23	—	—	9.9 (95% CI: 5.0–21.7)
Cured laminates/plates (tf > 0.45 mm)	106	14	—	—	4.8 (95% CI: 1.9–21.7)

Material efficiencies are expressed in $kN\cdot m\cdot kg^{-1}$.

S10. Subgroup predictive performance

Table S10-1 reports out-of-fold performance by FRP material, wrapping scheme, anchorage, and section type using the random ten-fold protocol.

Predictive performance was strongest among the major subsets for CFRP, U-jacketed systems, anchored systems, and T-sections. Performance was substantially weaker for the small GFRP, BFRP, and PET-FRP groups, where R^2 was negative or close to zero. Predictions within these sparsely represented material subsets should therefore be interpreted cautiously and should not be assumed to have the same reliability as those for the better-represented subsets. Anchored specimens achieved the highest subset-specific R^2 (0.848).

Table S10-1. Out-of-fold model performance by subset

**Subset**	n	R^2	**RMSE (kN)**	**MAPE (%)**	**Median APE (%)**
CFRP	291	0.806	30.4	63.1	27.1
GFRP	37	−0.447	12.4	58.3	33.9
AFRP	14	0.731	54.7	80.3	26.2
BFRP	7	−0.028	11.1	24.2	21.5
PET-FRP	10	0.090	37.7	67.7	29.7
Side-bonded	139	0.774	19.8	50.7	26.4
U-jacketed	159	0.817	34.5	73.0	29.3
Completely wrapped	61	0.743	37.9	62.6	30.7
Anchored	90	0.848	38.8	38.9	22.3
Unanchored	269	0.690	27.0	70.6	29.4
Rectangular section	210	0.759	26.7	47.3	26.7
T-section	149	0.826	35.0	84.1	32.2

The negative R^2 values for GFRP and BFRP indicate that, within those subsets, the out-of-fold predictions performed worse than a constant mean prediction based on the subset mean. These subgroup statistics should be interpreted cautiously because R^2 is sensitive to both sample size and within-subset response variance, particularly for the sparsely represented material classes.

S11. Excluded specimens and exclusion sensitivity

Of the 376 collected specimens, 17 were excluded from the principal analysis because the attributed FRP contribution, $V_{f,exp}$, was non-positive or could not be determined. The excluded records comprised 16 CFRP specimens and one GFRP specimen: one zero contribution, eight negative contributions ranging from −45.1 to −4.2 kN (all CFRP), and eight undetermined values (seven CFRP and one GFRP). The excluded specimens are listed in Table S11-1. Negative values arise from the difference between strengthened and companion-control capacities and may reflect experimental variability, premature debonding, adverse FRP–concrete interaction, or negligible mobilization. They are physically meaningful but cannot be represented on the logarithmic scale used for the principal positive-efficiency analysis.

Negative values of $V_{f,\mathrm{exp}}$ arise from the attributed difference between strengthened and companion control capacities and may reflect experimental variability, premature debonding, or negligible mobilization of the strengthening system. Because the adopted material-efficiency metric is defined only for positive values of $V_{f,\mathrm{exp}}$ and is evaluated on a logarithmic scale, non-positive values cannot be represented within the principal analysis. They were therefore excluded rather than assigned negative material-efficiency values.

Sensitivity to this exclusion was evaluated in two stages. First, all 17 excluded specimens were reintroduced as zero-efficiency observations; this deliberately conservative convention also assigns zero to the eight undetermined records and reduced the CFRP:GFRP median-efficiency ratio from 8.07 to 7.37. Second, the nine measurable non-positive observations (one zero and eight negative) were retained with their signed efficiencies, $V_{f,exp}/m$. Three signed-data summaries were evaluated with programme-clustered intervals: the ratio of signed medians was 7.51 (3.6–17.8); the pooled ratio of total attributed contribution to total installed mass was 7.88 (4.0–16.9), compared with 8.65 when the measurable non-positive observations were excluded; and a shifted-logarithm geometric-mean ratio, using a shift of 450 kN·m·kg^{-1}, was 8.04 (5.0–15.7), compared with 8.85 without them. Retaining adverse or negligible contributions therefore lowered the ratio modestly but did not materially change the break-even interpretation (Table S11-2).

Table S11-2. Sensitivity of the CFRP:GFRP efficiency contrast to non-positive and undetermined contributions.

Treatment / statistic	CFRP:GFRP ratio	Programme-clustered 95% CI
Principal positive-only median ratio	8.07	3.8–18.3
All 17 excluded records assigned zero	7.37	—
Signed medians; 9 measurable non-positive retained	7.51	3.6–17.8
Pooled total contribution / total mass; signed data	7.88	4.0–16.9
Pooled ratio with measurable non-positive excluded	8.65	—
Shifted-log geometric mean; signed data	8.04	5.0–15.7
Shifted-log geometric mean with measurable non-positive excluded	8.85	—

Table S11-1. Excluded specimens with non-positive or undetermined $V_{f,exp}$

Source	Specimen	FRP	Scheme	Section	$V_{f,\mathrm{exp}}$ (kN)	Failure mode
[10]	TR30D3	C	SB	R	0	Shear cracking with concrete-cover detachment; FRP contribution practically null
[12]	ST2b	G	CW	R	n.d.	Shear failure through concrete crushing on the compression side, accompanied by GFRP-sheet rupture
[15]	St-1LFRP-On	C	UJ	T	−4.5	Diagonal shear failure; CFRP strips aligned with the stirrups; debonding sequence not reported
[15]	St-2LFRP-On	C	UJ	T	−5.3	CFRP-strip debonding accompanied by diagonal shear failure
[17]	M2-a	C	UJ	T	−19.3	Shear failure with CFRP debonding
[17]	M2-b	C	UJ	T	−45.1	Shear failure with CFRP debonding
[19]	LB0.7U	C	UJ	T	−14	Fabric separation followed by shear failure
[19]	LB1.3U	C	UJ	T	−35	Fabric separation followed by shear failure
[19]	MB1.3U	C	UJ	T	−16	Fabric separation followed by shear failure
[32]	FSN-3	C	SB	T	−4.18	Shear failure with FRP delamination
[38]	L-S	C	UJ	T	n.d.	CFRP-strip rupture
[38]	L-S-W	C	UJ	T	n.d.	CFRP-strip rupture
[38]	L-S-I	C	UJ	T	n.d.	CFRP-strip rupture
[38]	H-S	C	UJ	T	n.d.	CFRP-strip rupture
[38]	H-S-R	C	UJ	T	n.d.	CFRP-strip rupture
[38]	H-S-M	C	UJ	T	n.d.	CFRP-strip rupture
[38]	H-S-2	C	UJ	T	n.d.	CFRP-anchor failure

n.d. = not determined; C = CFRP; G = GFRP; R = rectangular section; T = T-section.

S12. Environmental boundary matching

The break-even framework requires the database-derived material-efficiency ratio and the user-supplied emission-factor ratio to refer to the same product boundary.

The installed-mass term, m, represents the cured FRP composite sheet. The corresponding emission factor must therefore be expressed as kg CO_2e per kg of cured composite sheet. It should not correspond to dry fibre alone, resin alone, or a fully installed system that additionally includes adhesive, primer, labor, equipment, or transport.

The term “lower-GHG option” is used throughout the article rather than “carbon-preferable” to avoid ambiguity between greenhouse-gas performance and carbon-fiber reinforcement. The sheet-level criterion should not be interpreted as a life-cycle environmental preference without a full LCA that includes the excluded stages.

The direction of the most important boundary extension can nevertheless be bounded from the database. Adhesive, saturant, and primer demand scale primarily with bonded area rather than composite mass. Let q denote their cradle-to-gate GHG emission per square meter of bonded area, $A_i = (w_f/s_f)p$ the bonded area per meter of beam, and $AE_i = V_{f,exp}/A_i$ the attributed contribution per unit bonded area. The extended break-even condition adds an area-dependent term to the sheet-only criterion. In the database, median AE was 159 kN·m^{-1} for CFRP and 53 kN·m^{-1} for GFRP, giving a CFRP:GFRP area-efficiency ratio of 3.0 (programme-clustered 95% CI: 2.1–4.7; Table S12-2). Because AEGFRP < AECFRP, area-dependent consumables shift the break-even threshold in favor of CFRP relative to the sheet-only criterion; a sheet-level GFRP preference should therefore be confirmed using project-specific adhesive data. Service life acts multiplicatively: if system i requires N_i installations over the reference period, the threshold scales by NGFRP/NCFRP. End-of-life burdens per kilogram can be added to the material emission factor without changing the form of the sheet-level rule. Relative durability, replacement frequency, anchorage demand, and end-of-life routes were not reported consistently by the source programmes and therefore remain project-specific inputs.

Table S12-2. Attributed FRP contribution per unit bonded area.

Material	Median area efficiency, AE (kN·m^{-1})	CFRP:GFRP ratio
CFRP	159	3.0 (programme-clustered 95% CI: 2.1–4.7)
GFRP	53	—

Table S12-1. Cradle-to-gate boundary adopted for the installed-mass metric

Quantity	Included in *m*?	Boundary interpretation
Fiber production	Yes	Major burden and principal source of variation among fibre types
Matrix resin in the cured sheet	Yes	Included when the emission factor refers to the cured composite product
Sizing and fiber finishing	Yes	Included within the cured-composite-sheet emission factor
Adhesive or field-applied saturant	No	Application-dependent and primarily related to bonded area
Primer and putty	No	Site- and application-dependent consumables

Quantity	Included in $\boldsymbol{m}$?	Boundary interpretation
Installation energy and labor	No	Project-specific and outside the adopted environmental boundary
Transport to site	No	Project-specific
End-of-life treatment	No	Fibre-specific and appropriate for a whole-life comparison
Durability- or fatigue-related replacement	No	Requires service-life modelling outside the present scope
